# Epigenomic and transcriptomic signatures of a Klinefelter syndrome (47,XXY) karyotype in the brain

**DOI:** 10.4161/epi.27806

**Published:** 2014-01-29

**Authors:** Joana Viana, Ruth Pidsley, Claire Troakes, Helen Spiers, Chloe CY Wong, Safa Al-Sarraj, Ian Craig, Leonard Schalkwyk, Jonathan Mill

**Affiliations:** 1 University of Exeter Medical School; Exeter University; Exeter, UK; 2 Institute of Psychiatry; King’s College London; London, UK; 3 Garvan Institute of Medical Research; Sydney, NSW Australia

**Keywords:** Klinefelter syndrome, DNA methylation, gene expression, 47,XXY, prefrontal cortex, cerebellum

## Abstract

Klinefelter syndrome (KS) is the most common sex-chromosome aneuploidy in humans. Most affected individuals carry one extra X-chromosome (47,XXY karyotype) and the condition presents with a heterogeneous mix of reproductive, physical and psychiatric phenotypes. Although the mechanism(s) by which the supernumerary X-chromosome determines these features of KS are poorly understood, skewed X-chromosome inactivation (XCI), gene-dosage dysregulation, and the parental origin of the extra X-chromosome have all been implicated, suggesting an important role for epigenetic processes. We assessed genomic, methylomic and transcriptomic variation in matched prefrontal cortex and cerebellum samples identifying an individual with a 47,XXY karyotype who was comorbid for schizophrenia and had a notably reduced cerebellum mass compared with other individuals in the study (n = 49). We examined methylomic and transcriptomic differences in this individual relative to female and male samples with 46,XX or 46,XY karyotypes, respectively, and identified numerous locus-specific differences in DNA methylation and gene expression, with many differences being autosomal and tissue-specific. Furthermore, global DNA methylation, assessed via the interrogation of LINE-1 and Alu repetitive elements, was significantly altered in the 47,XXY patient in a tissue-specific manner with extreme hypomethylation detected in the prefrontal cortex and extreme hypermethylation in the cerebellum. This study provides the first detailed molecular characterization of the prefrontal cortex and cerebellum from an individual with a 47,XXY karyotype, identifying widespread tissue-specific epigenomic and transcriptomic alterations in the brain.

## Introduction

Klinefelter syndrome (KS) is the most common sex-chromosome aneuploidy in humans, affecting approximately 1 in every 600 newborn males.^1^ Most individuals with KS carry one extra X-chromosome (karyotype 47,XXY), although other reported variants include 48,XXXY, 48,XXYY and 49,XXXXY.^2^ The condition presents with a broad range of phenotypes that often vary in severity.^2^ In addition to the well-characterized physical and physiological features—tall stature, gynecomastia, hypogonadism, and absent spermatogenesis^3^—KS is also often associated with psychiatric and neurodevelopmental phenotypes including language-based learning disabilities, decreased verbal intelligence and difficulties with task planning and inhibitory control.^4^ Of note, individuals with KS frequently exhibit symptoms related to schizophrenia including schizotypal traits,^5^ auditory hallucinations^6^ and verbal cognition impairment.^7^ Furthermore, several structural brain abnormalities are associated with the disorder including abnormal cerebral asymmetry^8^^,^^9^ and reductions in the size of specific brain regions,^6^^,^^10^^,^^11^ total brain volume,^10^^,^^12^^,^^13^ and white matter volume.^9^^,^^10^

The mechanism(s) by which the supernumerary X-chromosome determines the phenotypes evident in KS are poorly understood, although skewed X-chromosome inactivation (XCI), gene-dosage dysregulation, and the parental origin of the extra X-chromosome have all been implicated,^14^^-^^16^ suggesting that epigenetic processes play an important role. However, little is known about the specific epigenetic changes associated with KS, especially in tissues relevant to the KS phenotype. A study comparing global long interspersed nucleotide element-1 (LINE-1) DNA methylation in whole blood from individuals with Turner’s syndrome (45,XO), healthy males (46,XY), healthy females (46,XX) and KS patients (47,XXY) reported that increased chromosomal number was associated with hypomethylation across the genome.^17^ Studies of genome-wide patterns of DNA methylation in patients with trisomy 21^18^ and trisomy 8^19^ also reveal large changes not limited to the supernumerary chromosome, indicating that widespread epigenetic changes may be a common feature of chromosomal aneuploidy. Moreover, differences in brain morphology have been associated with imprinting of the X-chromosome in Turner Syndrome, indicating that the parental origin of the X-chromosome may be important in mediating the psychiatric symptoms present in sex abnormalities.^20^

This study is the first to examine genome-wide patterns of DNA methylation and gene expression in two regions of the brain obtained post-mortem from a patient with a 47,XXY karyotype. We identify widespread tissue-specific epigenomic and transcriptomic alterations, providing potential clues about the molecular causes and consequences of KS.

## Results and Discussion

As part of an integrated “-omics” study of schizophrenia (Pidsley et al., submitted), we examined genome-wide patterns of DNA methylation, gene expression, and genetic variation in post-mortem cerebellum and prefrontal cortex brain tissue samples from schizophrenia patients and controls. During the standard quality control steps of these data we identified a discrepancy between reported and measured sex for one schizophrenia patient who displayed the genomic characteristics expected of both male and female samples simultaneously. As expected, across the entire set of samples, males and females showed distinct levels of DNA methylation across probes on the X-chromosome, with the exception of one sample recorded as male, who clustered with the female samples (Fig. S1A). This individual also clustered with females for *XIST* gene expression (Fig. S2A) but with males when DNA methylation and gene expression across the Y-chromosome were assessed (Figs. S1B and S2B). A 47,XXY karyotype was confirmed via the high-resolution SNP genotyping array data (Figs. S3 and S4) and the presence of the Y-chromosome was confirmed via a PCR-based sex-typing assay (Fig. S5). A number of other genomic alterations were identified in this individual (Table S1), although there was no obvious excess burden of autosomal copy number variations (CNVs), except for one region with four copies of a region spanning the *NKAIN2* locus. This is notable since copy number changes in this gene have previously been reported in neuropsychiatric phenotypes including neurodevelopmental disorders and schizophrenia.^21^^-^^24^ The patient’s autopsy report did not record KS, suggesting that the 47,XXY karyotype was undetected during the patient’s lifetime, although we did not have access to detailed pre-mortem medical records. In addition to schizophrenia, the autopsy report highlighted that the 47,XXY patient had a poor nutritional state, hepatomegaly, vascular spiders, nystagmus, dysdyadocokinesia, some degree of ataxia at the time of death, a known history of alcohol abuse and were prescribed the medications parentrocite and sulpiride.

Identifying structural brain abnormalities associated with a 47,XXY karyotype is of relevance given the established link between KS and several neuropsychiatric disorders including schizophrenia.^5^^,^^25^ Detailed records taken by the neuropathologist at autopsy show that although the 47,XXY patient had a similar total brain mass to other patients (47,XXY = 1417 g, all other samples = 1410 ± 182 g, other males = 1454 ± 201 g, females = 1325 ± 92 g) they had a markedly lower cerebellum mass (47,XXY = 111 g, all other samples = 170 ± 24 g, other males = 175 ± 27 g, females = 160 ± 15 g), equating to more than two standard deviations below the mean of the other samples (Fig. 1; Fig. S6). The reduced cerebellum mass is consistent with the patient’s autopsy report of movement disorders, and previous studies demonstrate an association between cerebellar ataxia and reduced cerebellar size.^26^ Reductions in cerebral volume and decreased cortical thickness in the left inferior frontal, temporal, and superior motor regions have been previously reported in KS,^13^ and a recent imaging study showed significant reductions in the volume of several brain regions, including the cerebellum, in KS patients compared with controls.^10^ Other studies have also described brain abnormalities in KS patients, including reductions in total brain volume,^12^ specific brain regions^6^^,^^11^ and white matter volume,^9^ as well as abnormal cerebral asymmetry.^8^^,^^9^ These latter findings are consistent with the theory that brain asymmetry and cerebral dominance in humans is determined by a XY homologous gene pair.^27^ Structural brain abnormalities have also been identified in patients with other types of X-chromosome aneuploidy^28^; a recent study reported increased cerebellum volume in Turner syndrome (45,XO) patients, contrasting with the decreased cerebellum volume in our patient (47,XXY).^20^ However, because reduced cerebellum mass has been associated with alcoholism^29^ it is possible that the reduction observed in the 47,XXY patient is related to the their alcohol abuse history.

**Figure:**
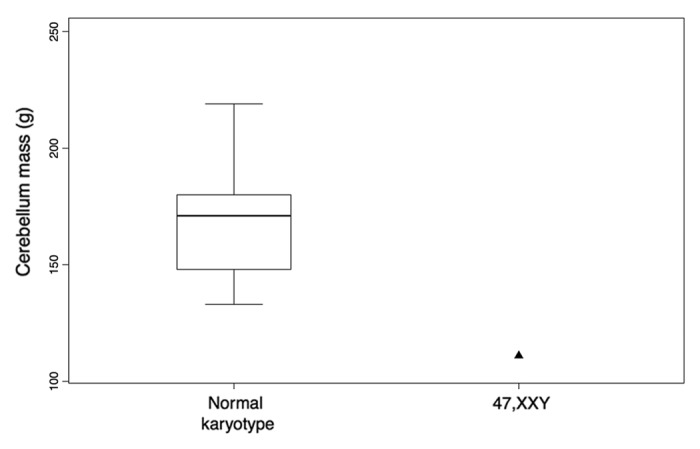
**Figure 1.** Reduced cerebellar mass in a 47,XXY patient comorbid for schizophrenia. Shown is the average cerebellar mass (in grams) across all samples compared with the cerebellar mass of the 47,XXY patient. Shown are the minimum, maximum, median and interquartile range of the cerebellar mass.

Global DNA methylation levels were estimated in both brain regions across all individuals using bisulfite-pyrosequencing assays targeting LINE-1 and Alu repeat elements across the genome, as described previously.^30^^,^^31^ Relative to other samples, the 47,XXY patient was a striking hypomethylated outlier across both LINE-1 (47,XXY = 67.7%, other samples = 73.0 ± 2.3%) and Alu (47,XXY = 25.5%, other samples = 28.1 ± 2.6%) repetitive elements in the prefrontal cortex (Fig. 2A and B**; **Fig. S7A and B). Conversely in the cerebellum, the 47,XXY individual showed notable hypermethylation compared with the other samples at LINE-1 repetitive elements (47,XXY = 78.4%, other samples = 71.9 ± 2.1%), although no significant difference was observed at Alu repetitive elements (47,XXY = 24.7%, other samples = 24.8 ± 0.8%) (Fig. 2C and D**;** Fig. S7C and D).

**Figure:**
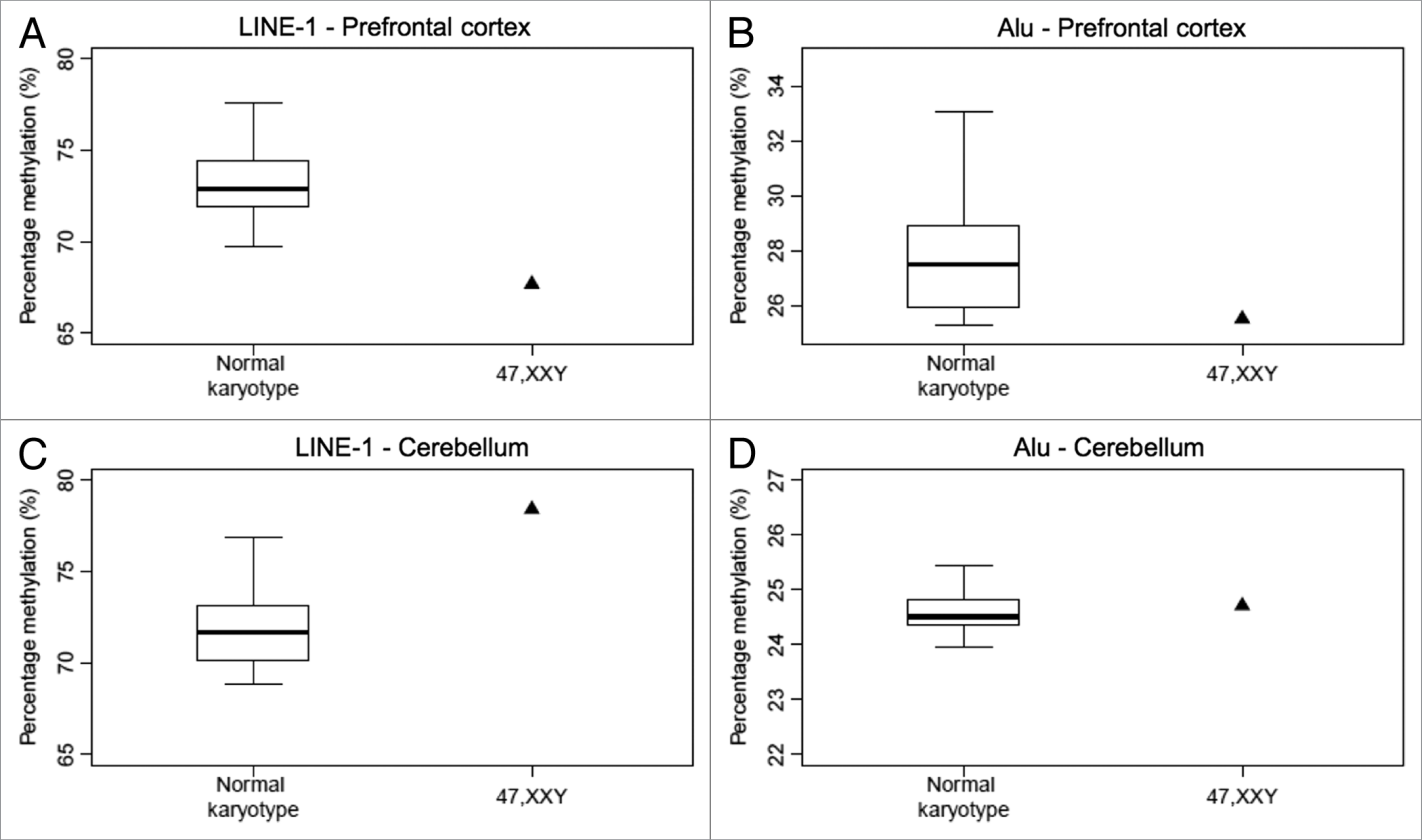
**Figure 2.** Tissue-specific differences in global DNA methylation in a 47,XXY patient comorbid for schizophrenia. The 47,XXY patient is significantly hypomethylated in the prefrontal cortex across both LINE-1 (**A**) and Alu repetitive elements (**B**) but hypermethylated in the cerebellum at LINE-1 elements (**C**), compared with the other samples. No significant difference was observed for DNA methylation across Alu repeat elements in the cerebellum (**D**). Shown are the minimum, maximum, median and interquartile range of the DNA methylation values.

Using the Infinium HumanMethylation450 BeadChip (Illumina Inc.) we identified numerous autosomal regions showing consistent differential DNA methylation in the 47,XXY patient compared with other samples (Tables 1 and 2). Of note, a region within the *sperm-associated antigen 1* (*SPAG1*) gene is significantly hypermethylated in both the prefrontal cortex and the cerebellum, an interesting observation in the context of the infertility associated with KS given the role of this gene in spermatogenesis, fertilization, and infertility.^32^ Other differentially methylated regions (DMRs) were found to be tissue-specific, and/or only detectable relative to either female or male controls. Of potential relevance to KS, for example, are cerebellar DMRs in the vicinity of *Piwi-like protein 1* (*PIWIL1*), which plays a role in the self-renewal of germline stem cells.^33^ Also of interest, given the cerebellar abnormalities seen in this individual, is evidence for cerebellum-specific hypermethylation of the *LIM/homeobox 4* (*LHX4*) gene; mutations in this gene have been associated with altered brain development and cerebellar structure.^34^ Given the comorbid diagnosis of schizophrenia in this patient, it is interesting that several of the prefrontal cortex DMRs are located in close proximity to other neurobiologically-relevant genes, including *NOTCH4, EPHB3* and *KCNN1*, that have been previously implicated in schizophrenia,^35^ brain development^36^^-^^38^ and regulation of microglia and neurons during neuroinflammation.^38^ The large differences in DNA methylation reported here are specific to the XXY individual; none of these regions are significantly differentially methylated in our analysis of schizophrenia and matched controls (Pidlsey et al., submitted).

**Table:** **Table 1.** 47,XXY-associated differently methylated regions in the prefrontal cortex

Gene	DMR position (hg19)	Probes in DMR	47,XXY vs. all other		47,XXY vs. other males		47,XXY vs. females	
			*β difference*	*P value*	*β difference*	*P value*	*β difference*	*P value*
VWA1	chr1:1374601–1374669	2			-0.05	6.28E-05		
	chr1:182669244–182669315	2					0.13	3.14E-06
	chr1:247681102–247681931	8			0.16	1.35E-06		
OR2L13	chr1:248100585–248100614	4					0.3	7.88E-06
	chr2:4931004–4931074	2	-0.23	2.07E-08	-0.23	3.66E-08	-0.23	3.93E-09
MAP4K3	chr2:39665105–39665186	2					-0.01	4.03E-05
PCBP1	chr2:70313772–70313833	2					0.01	2.57E-05
LOC388965	chr2:84517321–84517950	9			-0.03	3.14E-05		
CXCR1	chr2:219031640–219031719	2					-0.05	2.61E-05
IHH	chr2:219922729–219922998	2					0.02	6.33E-07
	chr2:240530497–240530569	2					-0.16	2.46E-05
WNT5A	chr3:55515168–55515541	3					0.03	2.18E-05
NEK11	chr3:130745442–130745959	13	0.04	7.82E-05			0.04	< 2.00E-16
DVL3	chr3:183887905–183888477	4					-0.05	4.49E-05
EPHB3	chr3:184297380–184297522	3			-0.07	1.33E-05		
	chr3:195578011–195578280	6					0.22	2.18E-05
	chr4:53588360–53588850	6					0.03	1.68E-06
SNHG8	chr4:119199621–119200372	11	0.04	2.30E-07	0.04	3.82E-11	0.04	2.16E-06
	chr5:784832–784915	3	0.5	< 2.00E-16	0.5	< 2.00E-16	0.5	< 2.00E-16
RIPK1	chr6:3077011–3077041	2					-0.16	9.23E-05
NOTCH4	chr6:32179862–32179971	2					-0.02	1.43E-05
TAPBP	chr6:33269769–33269832	2					-0.2	1.29E-08
	chr6:168120556–168120635	2			-0.06	3.07E-06	-0.05	1.94E-06
MAD1L1	chr7:2144559–2144767	3			-0.09	8.00E-07		
	chr7:157075207–157075303	2			-0.1	2.74E-05		
PTPRN2	chr7:157744316–157744347	2	-0.06	< 2.00E-16	-0.06	< 2.00E-16	-0.06	< 2.00E-16
	chr8:58055876–58056026	2	0.39	1.20E-05	0.4	2.33E-06	0.38	3.12E-05
C8orf71	chr8:58192753–58192883	2			-0.22	9.11E-05		
SPAG1	chr8:101224915–101225361	5	0.13	< 2.00E-16	0.13	< 2.00E-16	0.14	< 2.00E-16
SPAG1	chr8:101225800–101225902	2	0.22	< 2.00E-16	0.22	6.54E-12	0.23	< 2.00E-16
	chr10:2978126–2978687	5					-0.1	7.97E-09
	chr10:8090846–8090924	2					0.04	7.87E-05
STK32C	chr10:134062522–134062614	2					-0.21	1.95E-05
TMBIM4	chr12:66563381–66563928	10	0.03	3.12E-06	0.03	2.10E-07	0.03	4.13E-08
GALNT9	chr12:132904540–132904689	2			-0.11	3.24E-06		
NDRG2	chr14:21493406–21493410	2					0.03	2.15E-06
	chr14:95837801–95837929	3	-0.05	1.77E-06	-0.06	2.54E-06	-0.05	3.52E-07
LBXCOR1	chr15:68125566–68125599	2					-0.2	7.84E-06
	chr15:68126065–68126178	2					-0.04	8.16E-05
	chr19:17504632–17504972	3					0.15	2.61E-07
KCNN1	chr19:18077727–18077834	3					0.02	4.85E-08
AXL	chr19:41731934–41732589	5					0.03	3.79E-06
KDELR1	chr19:48894694–48895030	9	0.03	4.30E-05			0.03	3.79E-11
	chr21:44573854–44574022	3	-0.09	1.02E-07	-0.09	9.20E-08	-0.08	9.79E-10

Light gray boxes indicate non-significant results. Dark gray boxes indicate that no comparison was made (X-chromosome-linked genes were compared with females only whereas Y-chromosome-linked genes were compared with males only).

**Table:** **Table 2.** 47,XXY-associated differently methylated regions in the cerebellum

Gene	Position (hg19)	Probes in DMR	47,XXY vs. all other		47,XXY vs. males		47,XXY vs. females	
			*β difference*	*P value*	*β difference*	*P value*	*β difference*	*P value*
LHX4	chr1:180201891–180201893	2					0.26	5.42E-05
LHX4	chr1:180202256–180202784	2					0.09	2.92E-09
LHX4	chr1:180203135–180203143	8					0.05	1.49E-06
LAMB3	chr1:209799191–209799353	4	-0.14	7.94E-05			-0.14	3.66E-07
TTC15	chr2:3469154–3469529	2			-0.19	2.47E-07		
TET3	chr2:74328923–74329082	2			-0.07	5.17E-05		
	chr3:87137933–87138700	2	0.11	< 2.00E-16	0.11	< 2.00E-16	0.1	2.66E-12
CLDN18	chr3:137728810–137729296	9					-0.13	2.25E-05
NHEDC1	chr4:103940711–103941300	2			0.08	1.96E-07		
	chr5:784832–784915	2	0.43	< 2.00E-16	0.43	< 2.00E-16	0.43	< 2.00E-16
	chr5:68628240–68628738	2			0.1	3.90E-06		
RIPK1	chr6:3077011–3077041	3					-0.17	6.98E-05
HIST1H3C	chr6:26045532–26045663	13	0.1	3.02E-12	0.1	4.68E-09	0.1	< 2.00E-16
PTPRN2	chr7:157744316–157744347	4			-0.05	7.10E-10		
	chr8:58055876–58056175	3	0.43	8.50E-05	0.44	1.55E-06		
C8orf71	chr8:58191386–58192065	6	-0.25	4.89E-08	-0.26	3.82E-09	-0.25	1.38E-07
SPAG1	chr8:101224915–101225361	6	0.13	< 2.00E-16	0.13	< 2.00E-16	0.13	< 2.00E-16
SPAG1	chr8:101225800–101225902	11	0.21	< 2.00E-16	0.21	< 2.00E-16	0.21	< 2.00E-16
C9orf64	chr9:86571409–86572014	3	0.1	6.98E-05	0.1	6.25E-06		
KIAA1274	chr10:72254314–72254335	2					-0.11	1.70E-06
CALHM1	chr10:105218160–105218286	2					-0.1	6.26E-05
CALCB	chr11:15093613–15093769	2	-0.16	2.67E-06	-0.16	1.66E-06	-0.18	3.66E-07
TP53AIP1	chr11:128812804–128813442	2					-0.19	1.24E-05
TP53AIP1	chr11:128812846–128813008	3			-0.19	7.61E-05		
PIWIL1	chr12:130822286–130822818	2			-0.25	4.26E-05		
NBEA	chr13:36044860–36045352	2	0.05	3.26E-05	0.05	2.21E-06	0.05	8.17E-05
	chr15:26489846–26490045	2			-0.05	8.82E-05		
KRTAP17–1	chr17:39472114–39472340	2					-0.15	9.52E-05
	chr17:55213563–55213600	5					-0.15	9.68E-05
	chr17:77680078–77680232*	2	-0.19	6.73E-05			-0.19	3.31E-07
LRP5L*	chr22:25758621–25758749	5			-0.1	8.18E-05		
MXRA5	chrX:3264517–3265089	2					0.04	1.92E-09
TTTY14	chrY:21238886–21239607	2			0.09	1.18E-11		
* CNV with copy number of 3 overlapping with the region (see Table S1)								

Light gray boxes indicate non-significant results. Dark gray boxes indicate that no comparison was made (X-chromosome-linked genes were compared with females only whereas Y-chromosome-linked genes were compared with males only).

Numerous genes were found to be differentially expressed (DE) in the 47,XXY prefrontal cortex and cerebellum compared with other samples (Tables 3 and 4) with a Bonferroni-corrected z-score *P* value < 0.05. In both brain regions the vast majority of DE genes were characterized by increased expression in the 47,XXY patient (prefrontal cortex: 12 loci significantly upregulated, 1 locus significantly downregulated; cerebellum: 18 loci significantly upregulated, 0 loci significantly downregulated), suggesting that the supernumerary X-chromosome may be upregulating transcription at multiple autosomal loci across the genome. Although some DE genes were observed only in comparisons with both 46,XY males and 46,XX females, most were sex-comparison-specific. Strikingly, across both brain regions, many more 47,XXY DE genes were observed in comparison with females than males in both tissues (prefrontal cortex: 47,XXY vs females = 59, 47,XXY vs males = 18; cerebellum: 47,XXY vs females = 50, 47,XXY vs males = 21). Furthermore, although some changes were consistently observed in both the prefrontal cortex and cerebellum (e.g., *CAMP*, *EPCAM* and *LOC441208*), the majority were tissue-specific to a single brain region. The large differences in gene expression reported here are specific to the 47,XXY individual; none of these transcripts are differentially expressed in our analysis of schizophrenia and matched controls (Pidlsey et al., submitted).

**Table:** **Table 3.** 47,XXY-associated differentially expressed genes in the prefrontal cortex

nuID	Gene	Probe position (hg19)	Strand	47,XXY vs. all other		47,XXY vs. males		47,XXY vs. females		
				*fold change*	*P value*	*fold change*	*P value*	*fold change*		*P value*
fVedl_tOxJHgKDgkUk	ANGPTL7	chr1:11255703–11255752	+	0.17	1.11E-16	0.17	5.11E-15	0.17	< 2.00E-16	
Zl4IEsHlNCOIgeii9c	VCAM1	chr1:101204019–101204068	+					0.12	1.78E-08	
cqSF81At6xB7XyHjiQ	PRG4	chr1:186282872–186282921	+					0.2	< 2.00E-16	
lojns5Hg3o6bW_X07 g	CFH	chr1:196648872–196648921	+					0.23	1.43E-07	
Qo0T0_REI6OtlJLFOU	CFH	chr1:196658692–196658741	+					0.38	1.11E-16	
NIDSs_kv.U.0.u7g.8	H3F3A	chr1:226259333–226259382	+			0.33	7.90E-07			
NfqgAIRfuk8Lfn_vAk	EXOC8	chr1:231468728–231468777	-			0.21	1.68E-07			
ZoXhLA6oEHo3o160B8	EPCAM	chr2:47606943–47606992	+					0.69	1.11E-16	
BnrvPnvPuvuuMkvvno	EPCAM	chr2:47607046–47607095	+					1.04	2.26E-08	
cpAs4gp4jAojq4jkyo	EPCAM	chr2:47612328–47612349: 47613711–47613738	+	0.2	1.65E-09	0.2	2.98E-07	0.21	< 2.00E-16	
xd6Z60LRKEWCAU0bEY	EFEMP1	chr2:56149495–56149544	-			0.39	6.29E-10			
9l31SOkN7XN3uegjnI	AOX1	chr2:201535434–201535483	+					0.08	< 2.00E-16	
Zl7s26IBCIUH.09XlU	COL6A3	chr2:238232942–238232991	-					0.39	6.62E-14	
QXNDUD1d6L9O85e_Sk	COL6A3	chr2:238233095–238233144	-					0.13	6.77E-10	
rItcu7lcV6dKfep3iA	CAMP	chr3:48266905–48266954	+	0.2	< 2.00E-16	0.2	< 2.00E-16	0.2	< 2.00E-16	
69KffSiAkLEesX6HuQ	SLC25A20	chr3:48900042–48900091	-			0.36	5.69E-11			
lnxCeHgiilejtJhR.U	DNASE1L3	chr3:58179070–58179093: 58178505–58178530	-					0.13	< 2.00E-16	
6h.tJeHqxD3.vD_S7o	PHLDB2	chr3:111694722–111694770	+					0.27	3.50E-09	
ommprt5mtriFZmriGc	FAIM	chr3:138327937–138327986	+					0.11	3.54E-07	
ln0vn7fesf3vOp.6ok	TP63	chr3:189614936–189614985	+					0.21	< 2.00E-16	
KpJwqU0.HokiEUlMrU	NULL	chr4:144496759–144496808	-					0.09	5.31E-07	
35QRHIUd.o9CJHuzh4	GPX8	chr5:54460446–54460495	+					0.15	< 2.00E-16	
riWe5en0.0i4hP7Huk	BTNL9	chr5:180488062–180488111	+					0.2	3.57E-12	
QfyhEl_TCJbggfqkvk	LOC401233	chr6:3019504–3019553	-			0.15	7.79E-07			
fajeo6Sh6PMkUgoxnk	BMP5	chr6:55625255–55625272: 55623882–55623913	-	0.19	2.30E-14	0.18	7.69E-12	0.19	< 2.00E-16	
K6oCu8IOLU3SUMufrs	TBX18	chr6:85444495–85444544	-					0.12	6.56E-07	
07q5ey9ScdKKeKSiDg	IKBIP	chr6:168224491–168224540	-			-0.69	2.87E-07			
0uHF8y.yziXFfoICwo	TMEM196	chr7:19759195–19759244	-					0.21	4.12E-09	
Zn50ZUiIgAvCeeFeCk	INMT	chr7:30797037–30797086	+					0.12	3.31E-12	
cyxSgtKKIa_cDsvc3o	LOC441208	chr7:32768986–32769035	+	0.32	< 2.00E-16	0.33	< 2.00E-16	0.32	7.88E-15	
iX3qB7qexxL0p66.jk	FGL2	chr7:76825768–76825817	-					0.43	2.49E-11	
KmAXs1qVBeAE1SUIHo	COL1A2	chr7:94057677–94057726	+					0.35	< 2.00E-16	
9jUT7yueFyEiOB4rX4	COL1A2	chr7:94060111–94060160	+					0.45	< 2.00E-16	
QyCF6hIoee3ld3rRXk	DEFA1	chr8:6856660–6856709	-	0.12	1.38E-13	0.12	1.57E-12	0.12	1.11E-15	
xKBJBHXp4QcJQzpXK0	SCARA5	chr8:27727946–27727995	-					0.26	9.82E-07	
0LiHngASd5JSA633Ro	SDCBP	chr8:59492276–59492306	+					0.15	6.15E-07	
K7con83.RdfSKOc6rU	BNC2	chr9:16416697–16416746	-					0.17	1.83E-13	
6oXSUI4JlkkvBc4W5I	CCL19	chr9:34689927–34689940: 34689801–34689836	-					0.32	< 2.00E-16	
WrlIsC.cAwT13R_xVE	OGN	chr9:95146774–95146823	-					0.77	4.11E-15	
BDhFLxNahZPgiMZeio	OGN	chr9:95147978–95148027	-					0.88	< 2.00E-16	
ZjdFPOE1NVcnRRMkTI	OMD	chr9:95176881–95176930	-					0.45	3.73E-07	
Kc4qiig6iKqdKpQwHc	SLC27A4	chr9:131123437–131123486	+					0.09	3.26E-08	
oKAoV4glIpSToLgqkg	ITIH2	chr10:7788602–7788651	+					0.2	1.24E-09	
lqMskrfH4OAt4z_eOk	OTUD1	chr10:23730535–23730584	+					0.16	9.87E-11	
xqKT0lF6D5OJ2dL_o8	PDE6H	chr12:15134355–15134404	+					0.16	2.54E-08	
ZfKOiSBOiKCP0.wnXU	SLCO1C1	chr12:20905935–20905984	+			0.13	2.83E-10			
fX9eh..pR5XocuAg6E	PKP2	chr12:32944450–32944499	-					0.21	2.92E-07	
cjif7cfQC.v58VfSXU	GJB2	chr13:20761910–20761959	-					1	< 2.00E-16	
ciAGgiIoDiuq_igFTo	EDNRB	chr13:78470881–78470930	-	0.18	1.49E-07			0.18	1.11E-16	
xuigGSeQeCaIdwFSfk	PTGDR	chr14:52742862–52742911	+					0.15	< 2.00E-16	
rl55P5uN0IXUlILV9Q	PTGDR	chr14:52743020–52743069	+					0.32	< 2.00E-16	
0knRyVFXXc.6sIg.HE	TMEM30B	chr14:61744888–61744937	-					0.45	< 2.00E-16	
TP6d2kdUp6iejF5XpI	LOC388152	chr15:84871644–84871693	-					0.12	5.26E-09	
3eh0.Qkv5.70DbjiAU	NUDT21	chr16:56463489–56463538	-	0.15	1.04E-07	0.15	6.69E-08	0.15	5.92E-07	
KsuX1EryiLsDi3rL_0	TMEM220	chr17:10617182–10617231	-	0.09	2.07E-08	0.09	2.60E-09	0.08	3.79E-08	
x.Sd_F7Vd6eXeLeDdU	TOP2A	chr17:38545067–38545116	-	0.11	1.49E-07			0.12	< 2.00E-16	
EnpItS.SAMZeiUiS2E	FAM20A	chr17:66533566–66533615	-	0.12	2.70E-08	0.12	4.20E-09			
9k3mzbqMPhOKn4iB1I	CD177	chr19:43867372–43867421	+	0.14	6.18E-14	0.13	8.74E-12	0.14	< 2.00E-16	
3CBVEhgxeipOOJilWo	MYL9	chr20:35176437–35176486	+					0.11	1.34E-07	
No174RVAVBCigl6guU	SLPI	chr20:43882216–43882241: 43881769–43881792	-					0.27	< 2.00E-16	
ojLV_BETnlid6ABVEk	UBE2C	chr20:44444504–44444552: 44445348–44445348	+					0.1	< 2.00E-16	
r37yu690k8Sk.uedqI	LOC401397	chr20:57523176–57523225	-					0.11	2.69E-08	
TdSCif1KMn_KMJ5o4k	LOC96610	chr22:22664198–22664247	+					0.09	6.12E-08	
luuljtLxeOlOiCJCmE	ADRBK2	chr22:25817204–25817253	+					0.1	1.11E-16	
TADKWp66dGXsNUkf6Q	GGA1	chr22:38013841–38013890	+	-0.83	9.84E-07	-0.86	2.61E-09			
Z5K0omXVEuL9VRHadc	RRP7A	chr22:42907975–42908024	-					0.08	< 2.00E-16	
EqZLp0Zez1SR.qKUKQ	RRP7B	chr22:42969512–42969561	-					-0.55	6.55E-10	
0szegLje1eiuskL9Ro	NPM1	chrX:123414788–123414837	+			0.2	8.35E-08			
lLh40p.7RHpRI4TceU	GYG2P1	chrY:14518992–14518993: 14518689–14518736	-					0.11	1.21E-07	

Light gray boxes indicate non-significant results. Dark gray boxes indicate that no comparison was made (X-chromosome-linked genes were compared with females only whereas Y-chromosome-linked genes were compared with males only). Annotation data for each probe obtained using the Bioconductor package illuminaHumanv4.db.^39^

**Table:** **Table 4.** 47,XXY-associated differentially expressed genes in the cerebellum

nuID	Gene	Probe position (hg19)	Strand	47,XXY vs. all other		47,XXY vs. other males		47,XXY vs. other females	
				*fold change*	*P value*	*fold change*	*P value*	*fold change*	*P value*
uO7tSXg9R5ohX55GF4	TNFRSF18	chr1:1140794–1140843	**-**					0.15	1.01E-14
TQ5MLOicLl.6KP36CI	FAM76A	chr1:28087875–28087924	**+**					0.07	2.70E-07
3VHqE.9440_ek4J75o	PCNXL2	chr1:233275460–233275463: 233270891–233270936	**-**					0.14	6.48E-12
ZoXhLA6oEHo3o160B8	EPCAM	chr2:47606943–47606992	**+**					0.54	5.47E-12
BnrvPnvPuvuuMkvvno	EPCAM	chr2:47607046–47607095	**+**					0.9	3.44E-13
6KDbq30yk7941631Kg	KCNH7	chr2:163228344–163228393	**-**					0.12	7.42E-07
6R96hdG6D63d4rLojk	LOC401052	chr3:10048205–10048254	**-**					0.22	1.16E-10
TQeEon6idqJ_oip878	ARPP21	chr3:35722564–35722613	**+**					0.1	1.46E-12
rItcu7lcV6dKfep3iA	CAMP	chr3:48266905–48266954	**+**	0.09	3.46E-12	0.09	1.86E-09	0.1	< 2.00E-16
QEKvDrJSV50hXQ4Rno	ANKRD17	chr4:74005263–74005312	**-**					0.22	1.98E-07
0q44on0oRTyiSnoQTc	SH3TC2	chr5:148384360–148384409	**-**					0.1	5.29E-11
WVElhxxViAlxN9F9ec	RASGEF1C	chr5:179564876–179564899: 179564687–179564712	**-**			0.13	4.15E-07		
01Xd110F6gx9ITdQHQ	RNF39	chr6_mcf_hap5:1420068–1420117	**-**					0.16	6.29E-07
0cVOsh0SeXuuB.p1eU	PRRC2A	chr6_dbb_hap3:2890088–2890137	**+**					0.35	2.22E-07
Tqg7v6gLQi6yj6_nqY	C6orf132	chr6:42070437–42070486	**-**					0.23	3.15E-10
HqI33tUcoB5g_xRIqI	C6orf176	chr6:166338009–166338058	**-**					0.11	1.15E-08
cyxSgtKKIa_cDsvc3o	LOC441208	chr7:32768986–32769035	**+**	0.14	3.61E-07				
cyxSgtKKIa_cDsvc3o	LOC441208	chr7:32768986–32769035	**+**					0.15	< 2.00E-16
3WfZR.WSd6.5x7nndc	C7orf52	chr7:100813863–100813912	**-**	0.15	4.28E-09	0.15	1.03E-08	0.14	1.43E-09
K_N4laRVOKE5IkXkig	CNTFR	chr9:34552159–34552166: 34552028–34552069	**-**	0.23	< 2.00E-16	0.23	3.99E-14	0.23	< 2.00E-16
uJdPSKuSnCZOhiSaug	GPSM1	chr9:139252547–139252596	**+**					0.25	2.95E-08
90NSdfgVdSVn0l6k0c	ENTPD2	chr9:139942751–139942800	**-**					0.09	6.35E-08
3Z2Vqi1Jrte6J5ZTpU	IL15RA	chr10:6019449–6019498	**-**	0.08	8.68E-07			0.08	6.27E-08
ZIRQ6g1Q6oLY56590U	IGF2	chr11:2159459–2159460: 2156712–2156759	**-**	0.1	9.90E-14	0.09	1.45E-12	0.1	< 2.00E-16
Wl36k3qhE6w57l_Egs	CALCA	chr11:14988290–14988339	**-**	0.23	8.30E-09	0.23	5.73E-09	0.22	5.94E-09
x9f4JKzfk6VFquC1eU	TMEM223	chr11:62558209–62558258	**-**					0.28	6.22E-07
9ooIoDrhULuqvuvSgI	SLC22A6	chr11:62744143–62744192	**-**	0.18	3.65E-07	0.18	9.29E-07	0.18	9.39E-08
Z9IldVfociaepInc.BI	CNIH2	chr11:66051236–66051285	**+**	0.35	9.53E-07			0.36	4.88E-15
HFwXl7tTpgV3E1wXlo	SMARCC2	chr12:56558210–56558259	**-**	0.18	8.81E-08	0.19	4.36E-08	0.18	6.14E-07
QO_wesnjwsy9XkVfeo	LOC220115	chr13:53161055–53161104	**+**			0.12	8.32E-09		
c70LXLcyj6S.A5.HVU	OLFM4	chr13:53626107–53626156	**+**	0.09	4.37E-07	0.09	1.59E-07		
36JWb571RKl2v.XB_c	SPSB3	chr16:1826791–1826840	**-**					-0.89	9.17E-07
BloWN0NHoOuep7agcE	TNRC6A	chr16:24834917–24834966	**+**	0.13	9.96E-09	0.13	1.69E-08	0.13	9.43E-09
QLR0VHu.euUKd_KlUc	FAM64A	chr17:6354072–6354121	**+**					0.1	1.33E-07
EdOgRNRCeCkhClZIJ0	RPL19	chr17:37360385–37360427	**+**					0.7	6.11E-08
x6gXnXotNXeQEiJUi4	RBFOX3	chr17:77303806–77303842: 77231875–77231887	**-**					0.57	2.85E-09
iIoD3lFP9UreSVdSeo	RBFOX3	chr17:77303839–77303888	**-**					0.3	3.77E-07
f4oYbqlTz8YVp9fc6U	FN3K	chr17:80708373–80708422	**+**	0.22	1.29E-10	0.21	7.08E-10	0.23	1.34E-14
x3S.SNd5j.Pi6At_Z4	MGC70870	chr17_gl000205_random: 119141–119190	**+**					0.31	3.30E-07
oCVdUCZaWHZ7dnqh38	NFIC	chr19:3463515–3463564	**+**					0.1	1.67E-08
QudxDtFe.RtNJF3qhU	OLFM2	chr19:9964866–9964915	**-**	0.54	9.61E-07	0.57	1.20E-08		
9RSiJQISr7l8VSiUVc	NACC1	chr19:13251492–13251541	**+**	0.49	7.50E-11	0.49	1.49E-08	0.5	< 2.00E-16
uCKpSOEVwJKeNqQ6is	RAB3A	chr19:18307891–18307940	**-**					0.93	1.71E-12
l3roFeVfFJfjpJqDes	POU2F2	chr19:42595686–42595735	**-**					0.09	6.52E-08
T3DouuhUIkzS5yQDpI	UBE2V1	chr20:48700666–48700677: 48699413–48699451	**-**					0.19	8.02E-09
uXOOCKm1HogpVesKUk	KIAA1647	chr22:18958144–18958171	**+**					0.12	6.51E-07
i1_RF4d7R0Jf3UUpV0	SERPIND1	chr22:21141675–21141724	**+**					0.1	4.00E-07
Z5K0omXVEuL9VRHadc	RRP7A	chr22:42907975–42908024	**-**					0.16	4.44E-15
EU_ve.pMFc8DrmSJ4M	LOC389834	chrUn_gl000218:51064–51113	**-**			0.14	1.87E-07		
lt1Hf71dcOcPygIhR4	CHIC1	chrX:72903733–72903782	**+**					0.08	4.14E-06
WnUxEZ5faxUURBNQuk	L1CAM	chrX:153127445–153127494	**-**					0.77	1.28E-05
HLEukIKIKKaSqkOEnQ	L1CAM	chrX:153128160–153128209	**-**					0.08	2.23E-05
fKg7fXeNt9dKDIdCzk	RPS4Y1	chrY:2712151–2712200	**+**			1.48	< 2.00E-16		
Q8jADkAqS017VJIV90	NLGN4Y	chrY:16953254–16953303	**+**			0.06	1.27E-08		
6tUwTEFxS.3kFYCVKk	AL833666	chrY:21724080–21724129	**-**			0.18	3.20E-10		

Light gray boxes indicate non-significant results. Dark gray boxes indicate that no comparison was made (X-chromosome-linked genes were compares to females only whereas Y-chromosome-linked genes were compared with males only). Annotation data for each probe obtained using the Bioconductor package illuminaHumanv4.db.^39^

Given previous evidence that KS is associated with skewed XCI in peripheral blood,^14^ we next assessed allelic patterns of DNA methylation in the proximity of a polymorphic repeat (CAG)_n_ in the *androgen receptor* (*AR*) gene. Although the prefrontal cortex was characterized by subtle allelic imbalance of XCI (7.5% skewing), this did not exceed the range of normal skewing observed in our previous analysis of healthy individuals,^40^ and there was no evidence of allelic skewing in the cerebellum (0.4% skewing). We were also interested in examining the expression and DNA methylation status of X-linked genes believed to escape XCI in females.^41^ Between 5 and 15% of genes on the X-chromosome are thought to escape XCI in healthy females,^42^^,^^43^ and it has been suggested that these loci may play an important role in the KS phenotype.^44^^,^^45^ Although genes escaping XCI might be expected to show consistently high expression levels in females compared with males, many loci actually show variable levels of expression in females^46^ with evidence of tissue-specificity.^45^^,^^47^

Although the 47,XXY patient is an outlier for the expression of many genes thought to escape XCI, we find no consistent pattern of altered transcription; some loci are upregulated and others downregulated, with many showing changes specific to either male or female comparison groups (Table S2). There is also considerable evidence for tissue-specific differences in the expression of these genes, concurring with data from a study of genes escaping XCI in 41,XXY mice compared with karyotypically normal male and female mice.^45^ One of the loci showing noticeably higher expression in the 47,XXY patient compared with both females and males across both brain regions is the gene encoding Eukaryotic Translation Initiation Factor 1AX (*EIF1AX*) (Fig. S8), which has been suggested as a possible candidate gene for Turner syndrome (45,XO).^48^ Browseable tracks for viewing within the Integrative Genomics Viewer (IGV) (http://www.broadinstitute.org/igv/home) showing 47,XXY-associated changes in DNA methylation and gene expression for other loci are downloadable from our laboratory website (http://epigenetics.iop.kcl.ac.uk/XXY).

We also looked at the expression of genes located in the pseudoautosomal regions (PAR) 1 and 2^49^ (http://www.genenames.org/genefamilies/PAR), which are represented by three copies in individuals with a 47,XXY karyotype. Again, although often an outlier for transcription at these loci (Table S3), the observed pattern in the 47,XXY individual is heterogeneous, differing across tissues and between male and female comparison groups. Some PAR genes (e.g., *SLC25A6*) are clearly upregulated in the cerebellum but not the prefrontal cortex, while others (e.g., *DHRSX*) are upregulated across both tissues. Other genes such as *GTPBP6*, for example, are consistent outliers for reduced expression in the 47,XXY patient suggesting that transcription is not always positively correlated with copy number in the PAR. Further work is needed to explore the regulatory mechanisms influencing expression of loci on the extra X-chromosome, and the processes involved in controlling dosage compensation. Another region of interest in KS is the X transposed region (XTR) on the Y chromosome,^50^ created by a 3.5 Mb duplication from Xq21.3 to Yp11.2 during hominin evolution.^51^^-^^53^ In Figure S4 the allele frequency plot for the 46,XY male (**B**) shows three bands across both chromosomal regions, whereas the 47,XXY patient (**A**) is characterized by four bands, most likely resulting from cross hybridization of microarray probes. Although epigenetic deregulation of the *Protocadherin 11 X-linked* (*PCDH11X)* and *Y-linked* (*PCDH11Y)* genes in these regions are of great interest in KS and neuropsychiatric disease,^54^^-^^56^ DNA methylation array probes in the vicinity of these genes were excluded during our stringent quality control steps due to cross-reactivity^57^ and could not be assessed in this study (see the “Materials and Methods” section). Because it is plausible that the XTR contains sequence and epigenetic differences that are important in KS and schizophrenia, future studies should utilize methods that can unambiguously profile variation in this region.

In summary, this study identifies widespread transcriptomic and epigenomic changes in the prefrontal cortex and cerebellum associated with a 47,XXY karyotype. Although our findings are based on data from only a single 47,XXY individual, and it will be important to confirm the observed patterns samples from additional patients, this study represents the first detailed molecular characterization of brain tissue from an individual with a 47,XXY karyotype. The patient, who was comorbid for schizophrenia, was found to have a notably reduced cerebellum mass and was characterized by considerable locus-specific changes in DNA methylation and gene expression, with many of these differences being autosomal and tissue-specific. Strikingly, global DNA methylation, assessed via the interrogation of LINE-1 and Alu repetitive elements, was significantly altered in the 47,XXY patient in a tissue-specific manner. Finally, we find evidence for alterations in gene expression at loci believed to normally escape XCI in females.

## Materials and Methods

### Samples and nucleic acid isolation

Post-mortem brain samples were obtained from the MRC London Neurodegenerative Diseases Brain Bank (http://www.kcl.ac.uk/iop/depts/cn/research/MRC-London-Neurodegenerative-Diseases-Brain-Bank/MRC-London-Neurodegenerative-Diseases-Brain-Bank.aspx). Patients (n = 49) were approached in life for written consent for brain banking. All samples were dissected by a trained neuropathologist, snap-frozen and stored at -80°C following legal and ethical guidelines. The time between death and removal of the brain was recorded as post-mortem interval (PMI). The samples used in this study comprised of prefrontal cortex (PFC, n = 49) and cerebellum samples (CER, n = 48), from 23 schizophrenia patients (including the 47,XXY patient) and 26 unaffected controls. All schizophrenia patients were diagnosed pre-mortem by psychiatrists in the UK using standardized diagnostic criteria. Demographic information about the samples is summarized in Table S4. Genomic DNA was extracted from each tissue sample using a standard phenol-chloroform extraction and tested for purity and degradation using spectrophotometry and gel electrophoresis, respectively. RNA was extracted using a standard Trizol extraction method and purified using an RNeasy Mini Kit with DNase I digestion (Qiagen), according to manufacturer’s instructions. RNA was tested for degradation and purity using an Agilent 2100 Bioanalyzer and RNA 6000 Nano kit (Agilent Technologies). All samples were randomized with respect to gender and disease status throughout all stages of the project to avoid potential batch effects.

### Global DNA methylation assay

Bisulfite-PCR pyrosequencing was used to assess the methylation status of LINE-1 and Alu repeats as a proxy of global DNA methylation levels, as described previously.^30^^,^^31^ Samples were run on the Pyromark Q24 pyrosequencer (Qiagen) according to manufacturer’s instructions. DNA methylation levels for each sample were calculated as the average of the three interrogated CpG sites on each assay. Fully methylated and fully unmethylated control samples were included in all procedures to act as assay controls.

### Genome-wide DNA methylation array processing

500ng of genomic DNA from each sample was treated with sodium bisulfite in duplicate, using the EZ-96 DNA methylation kit (Zymo Research) following the manufacturer’s standard protocol. Duplicates were pooled and the samples (PFC n = 46 and CER n = 46) were assessed using the Illumina Infinium HumanMethylation450 BeadChip (Illumina Inc.) run on the HiScan System (Illumina Inc.). All samples were randomized with respect to gender and disease status to avoid batch effects, and processed on eight BeadChips.

### Methylomic data processing and analysis

Signal intensities were extracted using Illumina GenomeStudio software (Illumina Inc.) and imported into R^58^ using the *methylumi* and *minfi* packages.^59^^,^^60^ Multi-dimensional scaling plots of variable probes on the X- and Y-chromosome were used to check concordance between predicted and reported sex for each individual (see the “Results and Discussion” section). The comparison of non-CpG SNP probes on the array confirmed that the PFC and CER were sourced from the same individual where expected. Raw β values of CpG probes within brain region-specific differentially methylated regions (DMRs) (extracted from ref. 61) were used to confirm that the predicted and reported brain region corresponded for each sample. Probes containing a SNP with MAF > 5% within 10 bp of the CG target site based on the Illumina annotation data (n = 35 413) and non-CG probes (n = 65) were removed. Further stringent data quality control and processing steps were conducted using the *dasen* function in the *wateRmelon* package as previously described.^62^ The *pfilter* function was used to filter data by beadcount and detection *P* value to stringently control for poor quality probes (PFC n = 5623 probes and CER n = 10 417 probes removed across all samples). Prior to statistical analyses cross-reactive probes co-hybridizing to the sex-chromosomes, as previously identified,^57^ were removed. The *pnorm* function used to identify differentially methylated CpG sites in the 47,XXY patient and the *comb-p* package^63^ was used to identify 500bp regions of 2 or more adjacent differentially-methylated probes. The identified regions of differential DNA methylation were compared with copy number variation (CNV) data of the 47,XXY patient to screen for overlaps with any large genomic aberrations.

### Genome-wide expression array processing

An amount of 100 ng RNA from each sample (PFC n = 47 and CER n = 48) was biotinylated and amplified using the Illumina TotalPrep RNA Amplification kit (Life Technologies) to produce cRNA. cRNA was quantitated using a NanoDrop **NO-1000 (Thermo Fisher Scientific)** and RediPlate 96 RiboGreen RNA Quantitation Kit (Life Technologies). Genome-wide expression was assessed using the Illumina HumanHT-12 v4 Expression BeadChip (Illumina Inc.) according to manufacturer’s instruction.

### Expression data processing and analysis

Signal intensities for each probe were extracted using Illumina GenomeStudio software (Illumina Inc.) and imported into R using the *lumi* package within Bioconductor.^64^ Initial quality control checks using functions within *lumi* identified clear outlying samples, which were removed from subsequent analyses (PFC n = 5, CER n = 4). The sex of the samples was checked by comparing the sex predicted by the expression levels of the *XIST* gene with the reported sex for each individual (see the “Results and Discussion” section). Probes targeting transcripts of genes in the vicinity of brain region-specific DMRs^61^ were used to confirm that the predicted brain region corresponded with the reported region for each sample. Remaining samples were processed using the *lumi*^64^ and MBCB^65^ Bioconductor packages in R. During processing, probes with a detection *P* value > 0.01 across all samples were considered non-detectable and removed from subsequent analysis. The *ComBat* function within the *sva* package in R^66^ was used to adjust the data to remove batch effects. The *pnorm* function was used to identify differentially expressed transcripts in the 47,XXY sample. Genes identified as differentially expressed were compared with the CNV data of the 47,XXY patient to record overlaps with large genomic aberrations.

### Genome-wide CNV detection

200ng of genomic DNA from each prefrontal cortex sample were genotyped using the Illumina HumanOmniExpress BeadChip (Illumina Inc.). All samples were randomized with respect to gender and disease status to avoid batch effects. Illumina GenomeStudio was used to call genotypes (using the HumanOmniExpress-12v1_C.egt cluster file) with the default GenCall cut-off of 0.15. To compare the sex predicted by the genetic data with the reported sex for each individual previously published recommendations were followed.^67^ PLINK was used to assess the heterozygosity rate of the probes on the X-chromosome^68^ (see the “Results and Discussion” section). Autosomal CNVs were called using PennCNV.^69^

### PCR-based sex-typing assay

A PCR-based sex-typing assay was performed as described previously.^70^ In brief, the X and Y *amelogenin* (*AMELX*) sequences were amplified, with amplicons distinguished on the basis of size; the X-chromosome produces a 977bp amplicon, whereas the Y-chromosome produces a 788bp amplicon.

### X-Chromosome Inactivation assay

The allelic X-Chromosome Inactivation (XCI) ratios of both tissues of the 47,XXY sample were determined by assessing DNA methylation in the proximity of a polymorphic repeat (CAG)_n_ in the human *androgen receptor* (*AR*) gene, as described previously.^71^^,^^72^ In brief, 50 ng of genomic DNA was incubated with *Hpa*II, *Msp*I or water in triplicate. The digestion product was amplified using fluorescently labeled primers flanking the polymorphic repeat (CAG)_n_. An ABI3130 (Life Technologies) was used to separate the fluorescently labeled amplification products and quantify the peak heights of each allele. The XCI ratio was then calculated as previously described.^40^

## Supplementary Material

Additional material

Additional material

## Abbreviations:

KS: Klinefelter syndrome
XCI: X-Chromosome inactivation
LINE-1: long interspersed nucleotide element-1
PMI: post-mortem interval
CER: cerebellum
PFC: prefrontal cortex
DMR: differentially methylated regions
DE: differentially expressed
CNV: copy number variation
PAR: pseudoautosomal regions

## Notes

10.4161/epi.27806
