# Supplementary material for: Epigenomic and transcriptomic signatures of a Klinefelter syndrome (47,XXY) karyotype in the brain
Source: Epigenetics. 2014 Jan 29;9(4):587–99. doi: 10.4161/epi.27806 (PMC4121369; doi:10.4161/epi.27806)
Supplement: Additional material [file epi-9-587-s01.pdf]

Landes Bioscience www.landesbioscience.com

Supplemental Material to:

Joana Viana, Ruth Pidsley, Claire Troakes, Helen Spiers, Chloe CY Wong, Safa Al-Sarraj, Ian Craig, Leonard Schalkwyk, and Jonathan Mill

Epigenomic and transcriptomic signatures of a Klinefelter's syndrome (47,XXY) karyotype in the brain

Epigenetics 2013; 9(4)
http://dx.doi.org/10.4161/epi.27806

https://www.landesbioscience.com/journals/epigenetics/article/27806/

SUPPLEMENTARY ONLINE MATERIAL DESCRIPTION

Supplementary files include 8 supplementary figures (.pdf files) and 4 supplementary tables (.xlsx files). Additionally, it includes 4 track files created for viewing within the Integrative Genomics Viewer software (.igv files) which can also be downloaded from our laboratory website. The file names are listed below.

Supplementary file 1:

Figure S1. Multi-dimensional scaling plot of DNA methylation probes on the X-chromosome (A) and Y-chromosome (B). M=male, F=female. (**A**) Males and females show distinct X-chromosome methylation patterns, with the exception of a single male that clusters with the females (circled in red). (**B**) Males and females show distinct Y-chromosome methylation patterns. The same male patient (circled in red) here clusters with the males. (Note: the DNA methylation data from Y-chromosome probes in females is the result of background signal or Y-chromosome probes which show non-specific hybridization to autosomal regions. In subsequent analyses these probes were filtered out).

Supplementary file 2:

Figure S2. Plot of *XIST* gene expression (A) and mean Y-chromosome gene expression (B). M=male, F=female. (**A**) Males and females show distinct *XIST* expression levels, with the exception of a single male that clusters with females (circled in red). (**B**) Males and females show distinct Y-chromosome mean gene expression levels. The same male patient (circled in red) now clusters with other males. (Note: the expression levels from Y-chromosome probes in females are the result of background signal or Y-chromosome probes which show non-specific hybridization to autosomal regions. In later analyses these probes were filtered out).

Supplementary file 3:

Figure S3. Visualization of X-chromosome and Y-chromosome probe intensities for 47 prefrontal cortex samples run on the Illumina HumanOmniExpress BeadChip. The x-axis and y-axis represent the mean intensity (log R ratio) over all probes available on X-chromosome and Y-chromosome, respectively. XY males are represented by the blue triangles in the top left corner. XX females are represented by the red circles in the bottom right corner. The plot confirms one 47,XXY individual in the upper right corner.

Supplementary file 4:

Comparison of SNP probe intensities and allele frequencies between 47,XXY and a schizophrenia 46,XY patient. (**A**) Log R ratio and allele frequencies for the 47,XXY patient on the X- and Y-chromosomes. (**B**) Log R ratio and allele frequencies for a schizophrenia 46,XY patient on the X- and Y-chromosomes. The black lines delineate the Xq21.3 and Yp11.2 regions defining the XTR. The presence of the supernumerary X-chromosome on the 47,XXY patient leads to increased log R ratio on the X-chromosome compared to the 46,XY patient. The X-chromosome allele frequency plot of the 47,XXY patient shows heterozygous alleles clustered

around +0.5, whereas the same plot of the 46,XY patient shows just homozygous allele calls at 0 and 1. The log R ratio and allele frequency plots on the Y-chromosome are similar for both patients indicating just one Y-chromosome.

Supplementary file 5:

Figure S5. PCR-based sex-typing confirms the presence of the Y-chromosome in the 47,XXY patient. PCR-based sex-typing was performed as described previously[70], with X- and Y-chromosome amplicons distinguished on the basis of size; the X-chromosome produces a 977 bp amplicon, whereas the Y-chromosome produces a 788 bp amplicon. Shown are products from a 46,XX female (F), a 46,XY male (M) and the 47,XXY patient. L = 2-log DNA ladder (0.1-10.0 kb) (New England BioLabs Inc.).

Supplementary file 6:

Figure S6. Comparison of cerebellum mass (g) between the 47,XXY patient, females and males. The 47,XXY patient shows a significantly lower cerebellum mass compared to both female (F) and male (M) patients.

Supplementary file 7:

Figure S7. Comparison of global DNA methylation levels between the 47,XXY patient, females and males. The 47,XXY patient is significantly hypomethylated in the prefrontal cortex across both LINE-1 (**A**) and Alu repetitive elements (**B**) and hypermethylated in the cerebellum at LINE-1 elements (**C**) compared to females (F) and males (M). The difference is particularly evident when compared to females. The 47,XXY sample shows no significant difference in DNA methylation across Alu repeat elements in the cerebellum (**D**).

Supplementary file 8:

Figure S8. Visualization of gene expression differences at the Eukaryotic Translation Initiation Factor 1AX (*EIF1AX)* gene. The 47,XXY patient shows increased expression of the probes on the *EIF1AX* gene compared to males and females in both prefrontal cortex (PFC, green) and cerebellum (CER, red). This image was obtained using the Integrative Genomics Viewer software (Broad Institute, 2013). Browseable tracks for viewing differential expression and DNA methylation at other loci escaping XCI in IGV can be downloaded from our laboratory web-page (http://epigenetics.iop.kcl.ac.uk/XXY).

Supplementary file 9.xlsx:

Table S1. Autosomal CNVs detected in the 47,XXY sample.

Supplementary file 10.xlsx:

Table S2. Rank of the 47,XXY patient against other samples for transcription of probes associated with loci believed to escape X-chromosome inactivation (taken from Craig. [40]). Ranked from lower relative expression (lower numbers, red scale) to higher relative expression

(higher numbers, blue scale). Grey box = gene expression not detected in this tissue. Annotation data for each probe obtained using the Bioconductor package illuminaHumanv4.db47[73].

Supplementary file 11.xlsx:

Table S3. Rank of the 47,XXY patient against other samples for transcription of probes associated with loci residing in pseudoautosomal regions (PAR) 1 and 2 (http://www.genenames.org/genefamilies/PAR). Rank from lower relative expression (lower numbers, red scale) to higher relative expression (higher numbers, blue scale). Grey box = gene expression not detected in this tissue. Annotation data for each probe obtained using the Bioconductor package illuminaHumanv4.db47[73].

Supplementary file 12.xlsx:

Table S4. Demographic and sample information for all samples included in this study.

Supplementary file 13.igv:

IGV track file 1 - Beta value methylation difference (47,XXY-mean of all other samples) of genes escaping XCI in the cerebellum

Supplementary file 14.igv:

IGV track file 2 - **Beta value methylation difference (47,XXY-mean of all other samples)** of genes escaping XCI in the prefrontal cortex

Supplementary file 15.igv:

IGV track file 3 - Gene expression **difference (47,XXY-mean of all other samples)** of genes escaping XCI in the cerebellum

Supplementary file 16.igv:

IGV track file 4 - Gene expression **difference (47,XXY-mean of all other samples)** of genes escaping XCI in the prefrontal cortex

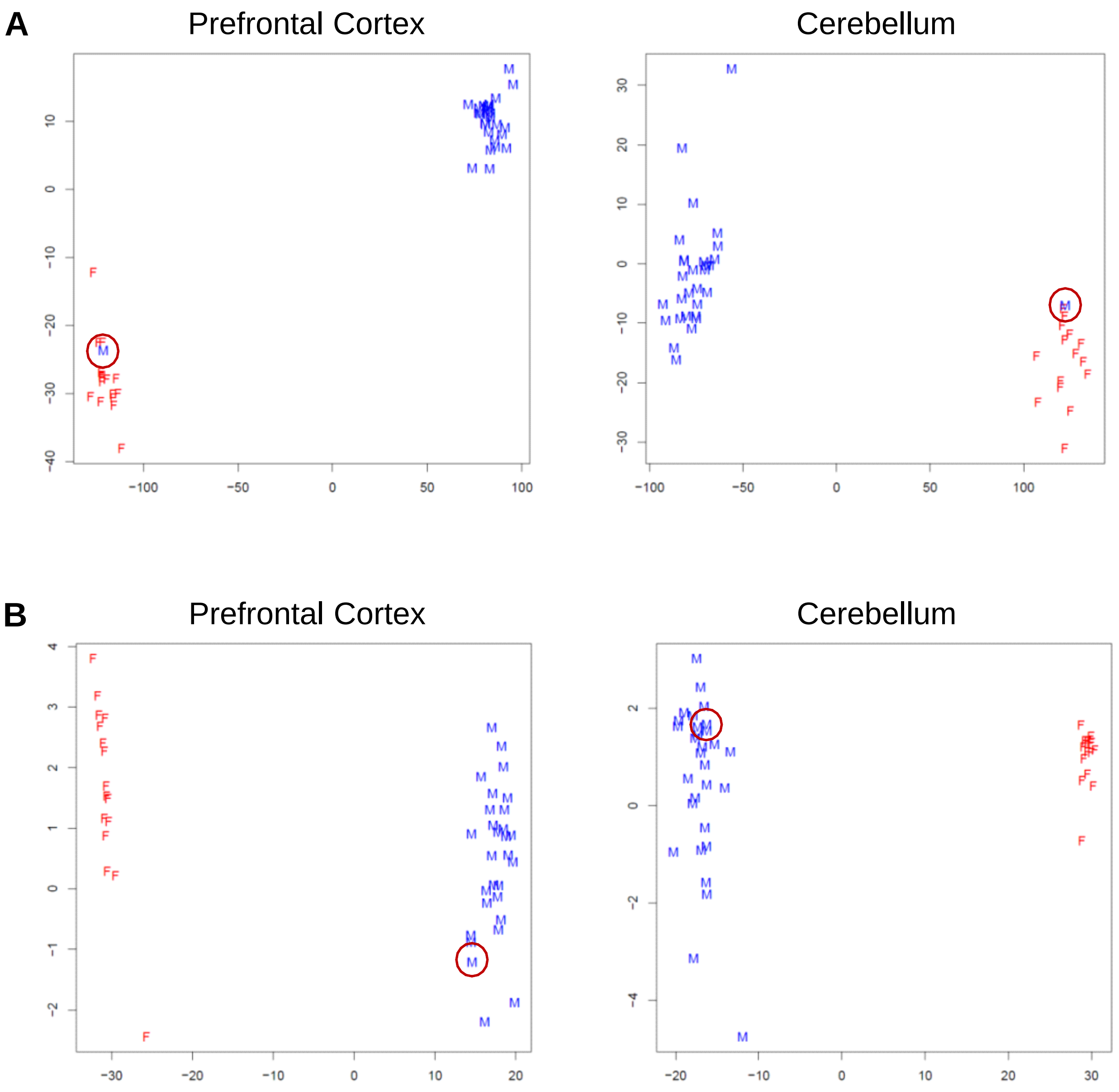
A
Prefrontal Cortex
Cerebellum
B
Prefrontal Cortex
Cerebellum

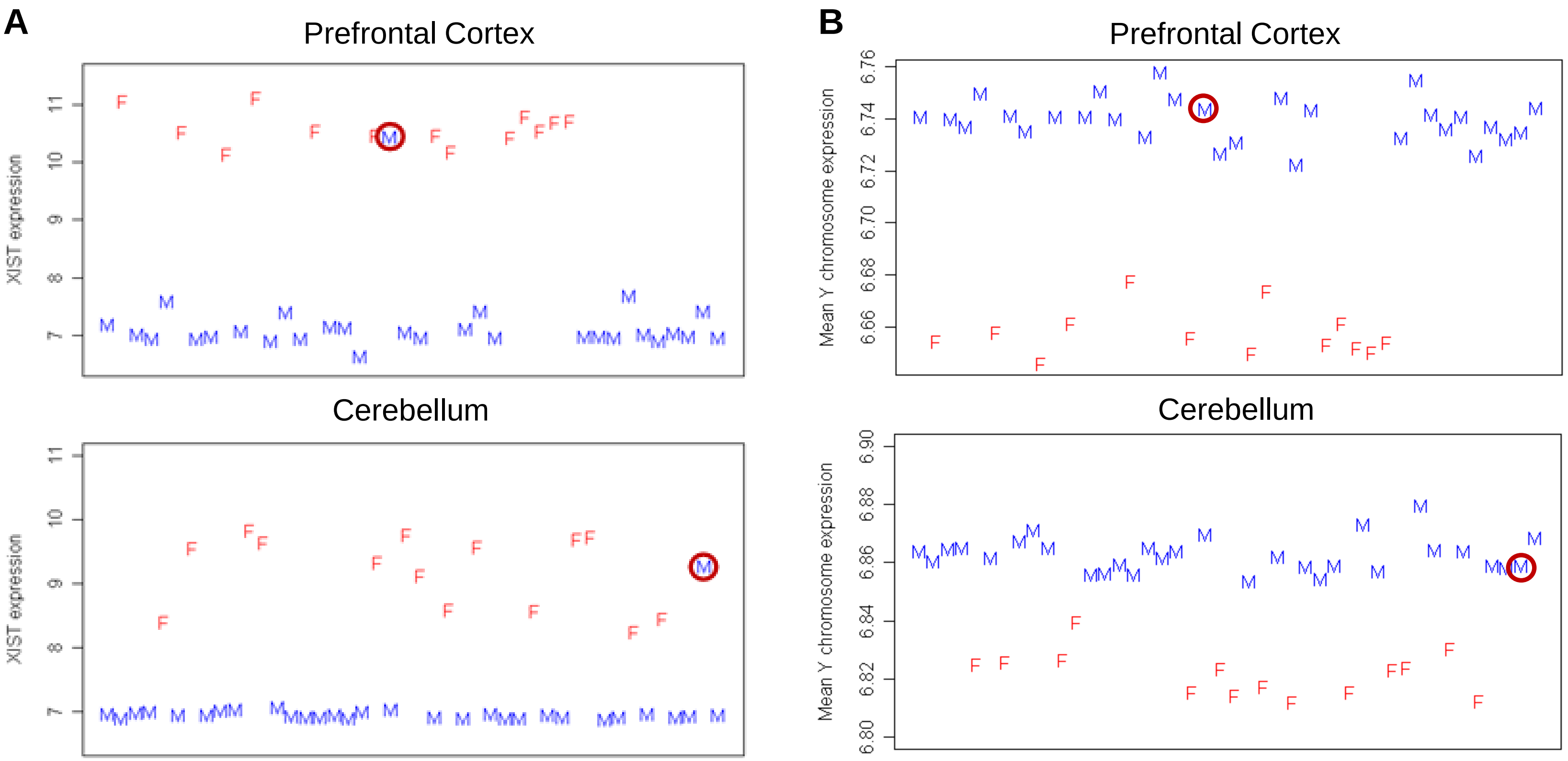
A
Prefrontal Cortex
XIST expression
7
8
9
10
11
Cerebellum
XIST expression
7
8
9
10
11
B
Prefrontal Cortex
Mean Y chromosome expression
6.66
6.68
6.70
6.72
6.74
6.76
Cerebellum
Mean Y chromosome expression
6.80
6.82
6.84
6.86
6.88
6.90

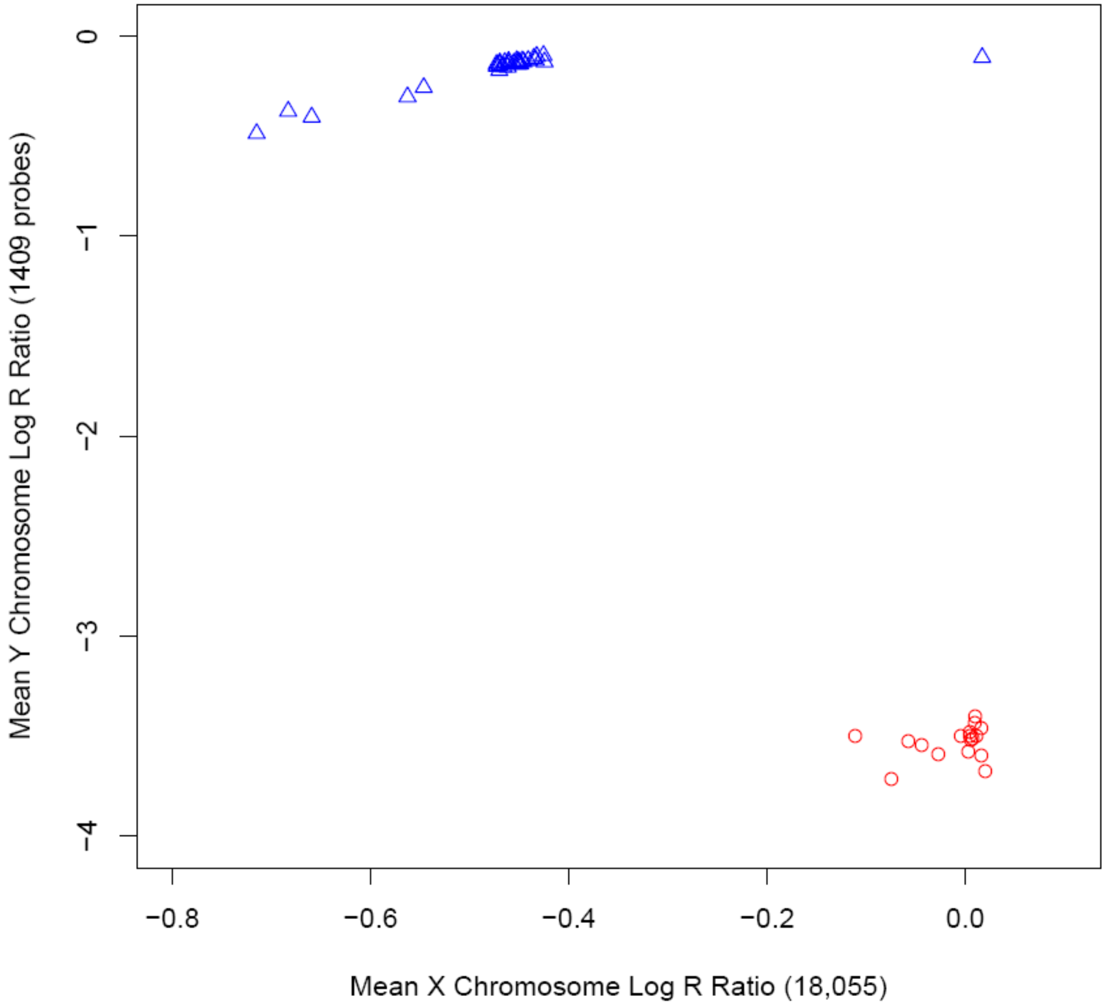
0
−1
−2
−3
−4
Mean Y Chromosome Log R Ratio (1409 probes)
−0.8
−0.6
−0.4
−0.2
0.0
Mean X Chromosome Log R Ratio (18,055)

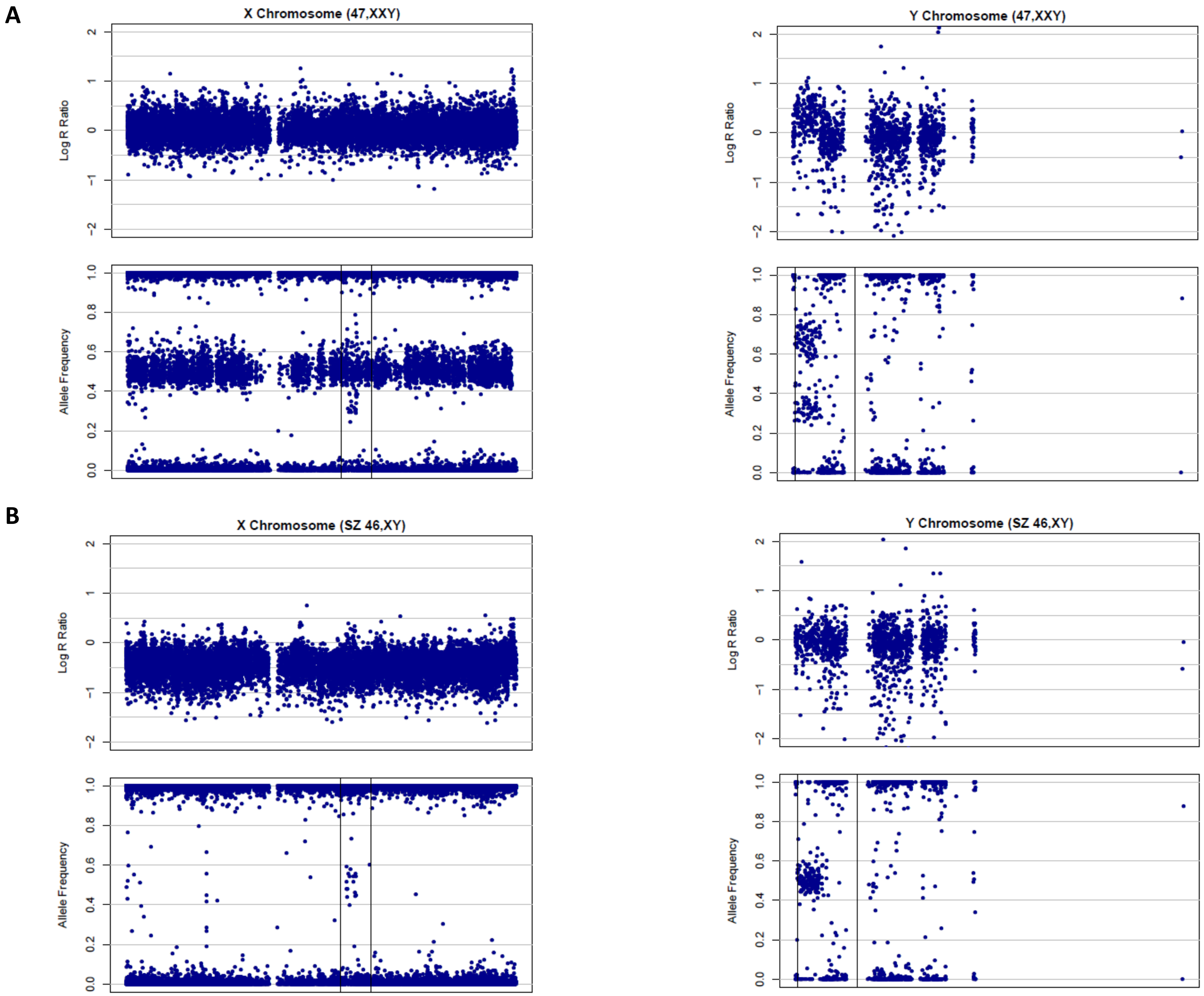
A
X Chromosome (47,XXY)
Y Chromosome (47,XXY)
Log R Ratio
Allele Frequency
B
X Chromosome (SZ 46,XY)
Y Chromosome (SZ 46,XY)
Log R Ratio
Allele Frequency

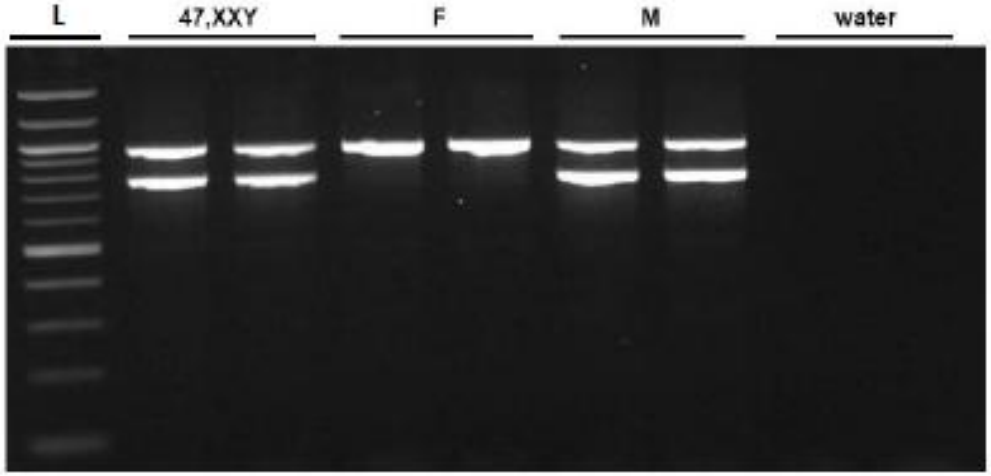
L
47,XXY
F
M
water

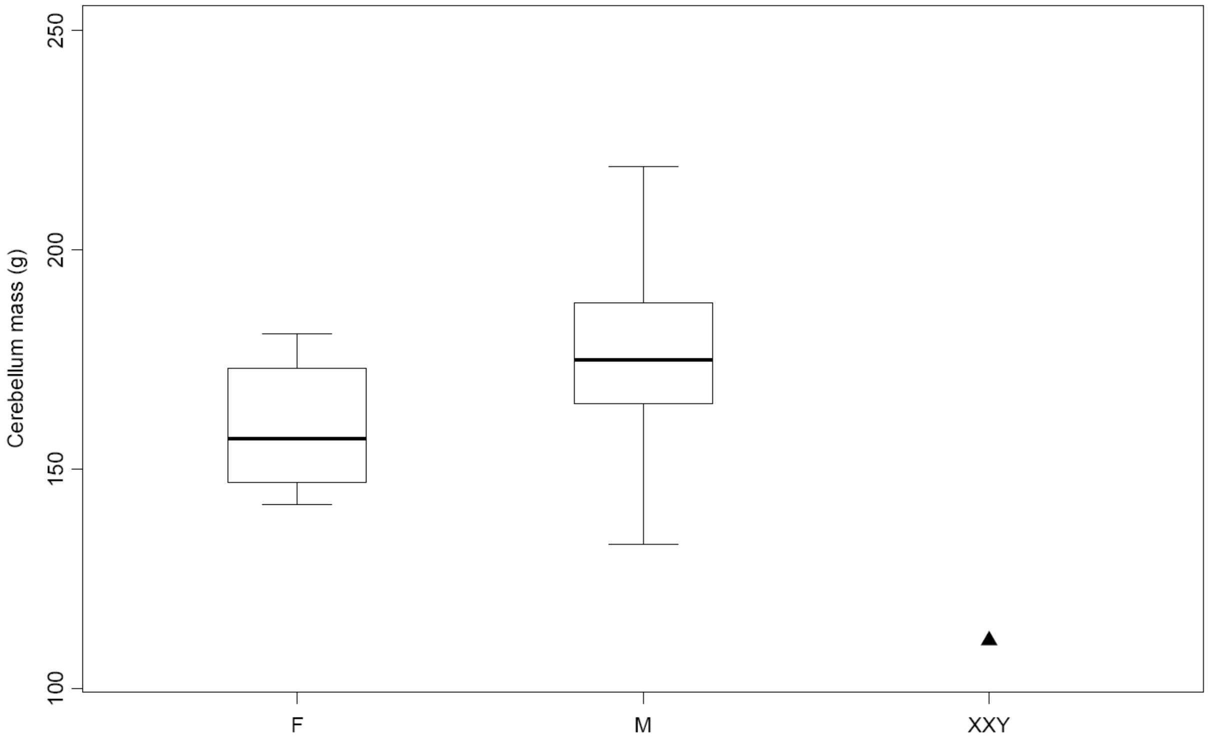

Cerebellum mass (g)
250
200
150
100
F
M
XXY

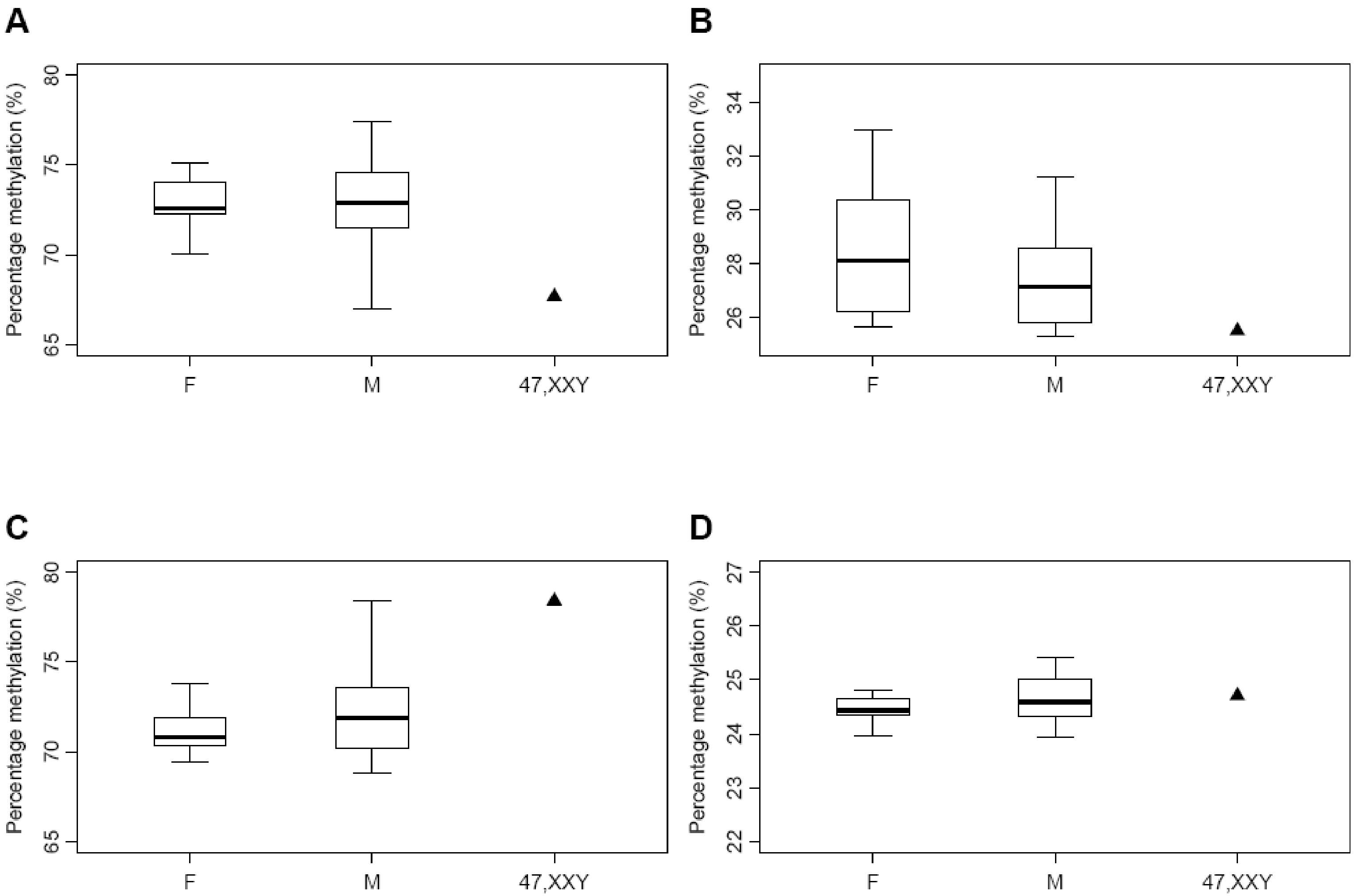
A
B
C
D
Percentage methylation (%)
80
75
70
65
34
32
30
28
26
27
25
24
23
22
F
M
47,XXY

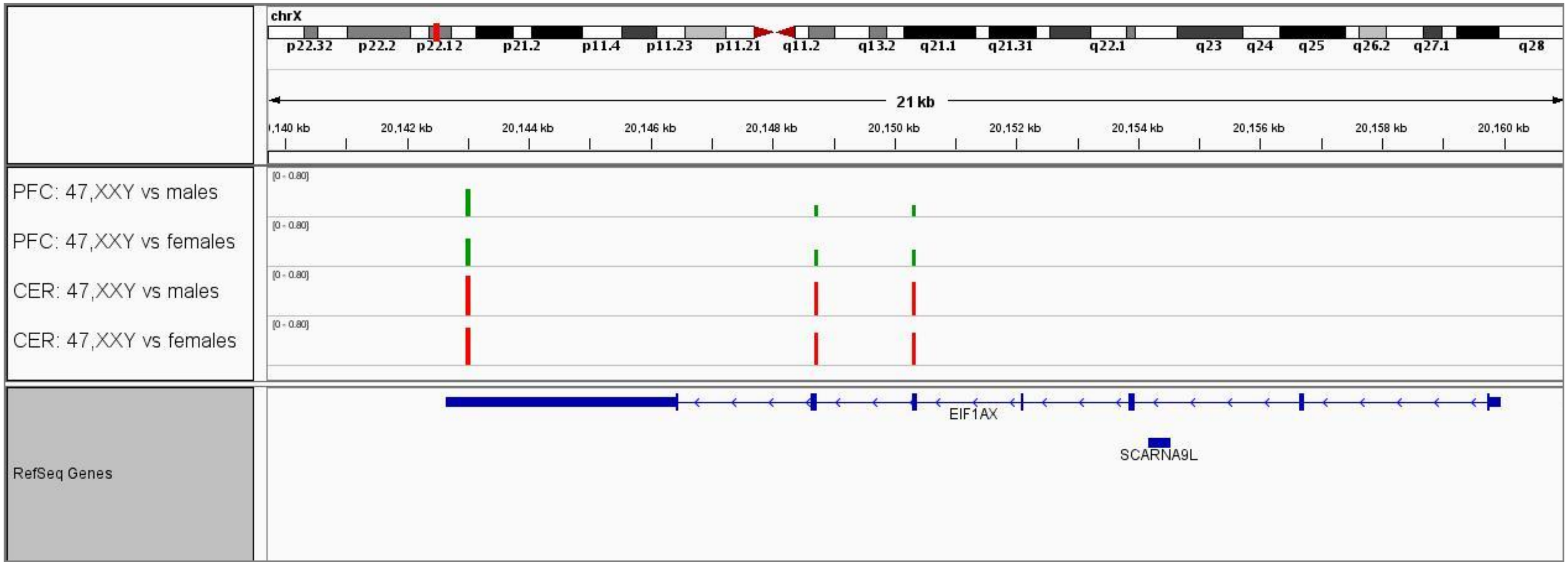
chrX
p22.32
p22.2
p22.12
p21.2
p11.4
p11.23
p11.21
q11.2
q13.2
q21.1
q21.31
q22.1
q23
q24
q25
q26.2
q27.1
q28
21 kb
,140 kb
20,142 kb
20,144 kb
20,146 kb
20,148 kb
20,150 kb
20,152 kb
20,154 kb
20,156 kb
20,158 kb
20,160 kb
PFC: 47,XXY vs males
PFC: 47,XXY vs females
CER: 47,XXY vs males
CER: 47,XXY vs females
[0 - 0.80]
RefSeq Genes
EIF1AX
SCARNA9L

Table S1. Autosomal CNVs detected in the 47,XXY sample.

Region (hg19)	No. of probes	*Copy #	Genes
chr2:208355809-208357838	7	0	
chr4:951179-1045265	30	3	TMEM175, DGKQ, SLC26A1, IDUA, SLC26A1, FGFRL1
chr4:10396709-10400156	6	0	
chr5:97047653-97099320	19	1	
chr5:115540041-115601291	15	1	COMMD10
chr5:140165568-140261235	40	3	PCDHA1, PCDHA2, PCDHA3, PCDHA4, PCDHA5, PCDHA6, PCDHA7, PCDHA8, PCDHA9, PCDHA10, AX746964, PCDHA11, PCDHA12
chr5:140504310-140532409	31	3	PCDHB4, PCDHB5, PCDHB6
chr5:140723174-140822723	54	3	PCDHGA1, PCDHGA2, PCDHGA3, PCDHGB1, PCDHGA4, PCDHGB2, PCDHGA5, PCDHGA9, PCDHGB3, PCDHGA6, PCDHGA7, PCDHGA8, PCDHGB4, PCDHGA8, PCDHGB5, PCDHGB6,PCDHGA10, PCDHGB7, PCDHGA11, PCDHGB8P, PCDHGA12
chr6:121746470-121759439	23	3	GJA1
chr6:124434003-124468892	10	4	NKAIN2
chr7:44935-68920	13	3	
chr10:106031030-106033903	9	3	GSTO2
chr13:100615239-100648099	13	3	ZIC5, ZIC2
chr14:106326623-106949307	12	3	DKFZp686O16217, IGHE, IGHG1, IGHD, FLJ00382, FLJ00382, AK128652, KIAA0125, ADAM6, BC042994, LINC00226, Z49973, BC011773, LINC00221
chr17:34458934-34476396	14	3	
chr22:25658525-25835514	78	3	BC040576, IGLL3P, LRP5L

*PennCNV copy number

Table S2. Rank of the 47,XXY subject against other samples for transcription of probes associated with loci believed to escape X-chromosome inactivation (taken from Craig. [40]). Rank from lower relative expression (lower numbers, red scale) to higher relative expression (higher numbers, blue scale). Grey box = gene expression not detected in this tissue. nuID for each probe obtained using the Bioconductor package illuminaHumanv4.db [73].

nuID	Gene	Probe coordinates (hg19)	Strand	Prefrontal cortex 47,XXY rank all	Prefrontal cortex 47,XXY rank males	Prefrontal cortex 47,XXY rank females	Cerebellum 47,XXY rank / all	Cerebellum 47,XXY rank males	Cerebellum 47,XXY rank females
				n=41	*n=29*	*n=13*	*n=43*	*n=29*	*n=15*
WO_UG596pV7R5Z6OWY	PRKX	chrX:3522660-3522709	-	8	7	2	9	7	3
lQF86X5FWSBLV9cMko	PRKX	chrX:3522945-3522994	-	6	5	2	13	13	1
WnSV9cql3lf47X4nHc	HDHD1	chrX:6967201-6967250	-	38	29	10	24	21	4
9ni1Ld3VccQtVPnutU	STS	chrX:7272357-7272406	+	19	15	5	37	27	11
lkTARQY6Xf.ii85MhU	PNPLA4	chrX:7867185-7867234	-	41	29	13	39	26	14
6vSOEmFl.09VWUXsdE	TBL1X	chrX:9687610-9687659	+	8	8	1	10	8	3
ZVSIZ0n7NT4egt7Ruk	CLCN4	chrX:10203332-10203381	+	25	16	10	23	17	7
WCCZ5K_Xruh3t8ApdU	MSL3	chrX:11778975-11779024	+	10	9	2	19	12	8
raSShE1lJOVCd9eReg	MSL3	chrX:11783794-11783843	+	23	14	10	11	7	5
WktiXqYRAiFFe3jIwc	MSL3	chrX:11785722-11785771	+				18	10	9
HVRNhyS8dP591c7VeE	MSL3	chrX:11785862-11785911	+	3	2	2	13	10	4
TnqJL5KUS4Lu_fe0fw	TRAPPC2	chrX:13730737-13730786	-	37	27	11	21	17	5
0Sk4FFuVpSgwn.h8VA	TRAPPC2	chrX:13731262-13731311	-				2	1	2
TUjtd_gkI2daviNRIk	TRAPPC2	chrX:13752206-13752255	-				23	17	7
x3EuAic1bfV.EHt4H8	CA5B	chrX:15802415-15802464	+	29	21	9	36	24	13
xpEPRgkiggMqFkkqFk	ZRSR2	chrX:15841166-15841215	+	5	5	1	11	11	1
BuPuFBPSlG4ke3zRNk	AP1S2	chrX:15844225-15844274	-	20	13	8	40	27	14
fueRH8SSfX9.U6R5cs	AP1S2	chrX:15844477-15844526	-	30	19	12	34	22	13
ZtGE1geioQo3wFQLGI	RBBP7	chrX:16863144-16863193	-	19	13	7	30	19	12
r1J9VFJV1CuuO60Q2o	CRLF2	chrX:17897216-17897231	+				14	10	5
NVP3.m5Lf49sQyCgRs	EIF1AX	chrX:20142985-20143034	-	41	29	13	43	29	15
BKTGmJ9SJOcA0OAeMQ	EIF1AX	chrX:20150300-20150326:<br>20148703-20148725	- -	37	25	13	43	29	15
ueEgke2dzggSeqZez4	SMS	chrX:22010730-22010779	+	29	19	11	39	25	15
ike2dzggSeqZez7Xug	SMS	chrX:22010739-22010788	+	27	17	11	26	17	10
391X5316XgEagFItAI	EIF2S3	chrX:24095795-24095844	+	29	25	5	41	29	13
TruB1JUDlLQLQpOr.U	ZFX	chrX:24231696-24231745	+	38	29	10	33	26	8
ijlhSBROLSDTSDNUUE	RPGR	chrX:38128919-38128968	-	39	28	12	27	20	8
upArHT6l0v303cA6Lo	RPGR	chrX:38144030-38144079	-				24	14	11
BeSKHc0kbOaYIiKiK0	RPGR	chrX:38158209-<br>38158250:38156698-38156705	- -	31	22	10	9	8	2
fVLhugtNIFUax_ixd0	USP9X	chrX:41092817-41092866	+	41	29	13	43	29	15
T.V1A.MrsIgmqkK5FE	USP9X	chrX:41095608-41095657	+	36	27	10	41	28	14
rSFab6S7JTB.eO8sAE	DDX3X	chrX:41208966-41209015	+	23	19	5	32	23	10
ZpLd0AtMeUi3cqQogE	FUNDC1	chrX:44383336-44383385	-	34	24	11	22	15	8
HqIix51x1KHdECeOJ4	KDM6A	chrX:44970808-44970857	+	34	25	10	31	26	6
QGGImmKO2K9XO1jEU0	UBA1	chrX:47074273-47074322	+	40	29	12	25	17	9
Hl63olXNeRXec7e47Q	SYP	chrX:49044434-49044483	-	19	12	8	43	29	15
fwygeCZaiU4J5KFTik	SMC1A	chrX:53401356-53401405	-	12	9	4	28	18	11
9jlZFNZxVjVdNCuDjE	RPS4X	chrX:71493707-71493756	-	38	29	10	23	19	5
N7v5121NRa1UQviIu0	RPS4X	chrX:71496007-71496013:<br>71495532-71495574	- -	40	28	13	33	24	10
3tAR_4f_fw9Tg715dc	ACSL4	chrX:108884977-108885026	-	41	29	13	30	20	11
oACtCleeoqDtZOOe3o	ACSL4	chrX:108911443-108911492	-	18	15	4	15	10	6
3l_nsec98eefc8_X30	ACSL4	chrX:108926784-108926833	-				10	6	5
ZirTd9z7F5JGfXqe8Q	ALG13	chrX:110928303-110928331:<br>110931115-110931135	+ +				27	20	8
uKHxz7.8KN13RLrDgE	ALG13	chrX:110931892-110931941	+	21	16	6	31	21	11
ip06xe99zUBf0PTnF4	VAMP7	chrX:155172999-155173048	+	29	19	11	36	25	12

Table S3. Rank of the 47,XXY subject against other samples for transcription of probes associated with loci residing in pseudoautosomal regions (PAR) 1 and 2 (http://www.genenames.org/genefamilies/PAR). Rank from lower relative expression (lower numbers, red scale) to higher relative expression (higher numbers, blue scale). Grey box = gene expression not detected in this tissue. nuID for each probe obtained using the Bioconductor package illuminaHumanv4.db [73].

		Probe coordinates (hg19)				Prefrontal cortex			Cerebellum		
**nuID**	**Gene**	**Chromosome X**	**Chromosome Y**	**PAR**	**Strand**	**47,XXY rank all**	**47,XXY rank males**	**47,XXY rank females**	**47,XXY rank / all**	**47,XXY rank males**	**47,XXY rank females**
						n=41	*n=29*	*n=13*	*n=43*	*n=29*	*n=15*
rsVr5xrgOCuVZNSAhk	PLCXD1	chrX:219773-219822	chrY:169773-169822	1	+	33	21	13	29	17	13
f1LqV2uXnuB5.V2g78	GTPBP6	chrX:221456-221505	chrY:171456-171505	1	-	1	1	1	2	2	1
iJ3aHqILGaWKLGE164	PPP2R3B	chrX:299374-299423	chrY:249374-249423	1	-	28	17	12	31	18	14
BcbTceQveonoRoUYXk	PPP2R3B	chrX:306290-306339	chrY:256290-256339	1	-	34	23	12	41	28	14
lW5K4ToOUkDHUoUF4c	CRLF2	chrX:1327710-1327759	chrY:1277710-1277759	1	-				8	5	4
HPRVIoKgKcWYIrfhbg	CSF2RA	chrX:1428309-1428358	chrY:1378309-1378358	1	+	36	25	12	25	17	9
Z9FUigqApxZgit_FuA	CSF2RA	chrX:1428310-1428359	chrY:1378310-1378359	1	+	39	27	13	35	23	13
QW4por7LiUI2RT5EUE	CSF2RA	chrX:1428704-1428753	chrY:1378704-1378753	1	+	20	15	6	17	13	5
ZStuSAH4h6r0qfuqt4	IL3RA	chrX:1501338-1501387	chrY:1451338-1451387	1	+	29	21	9	18	12	7
cZNeXsjpaoKdyAKpk8	SLC25A6	chrX:1505329-1505378	chrY:1455329-1455378	1	-	20	10	11	43	29	15
xOU35RQLpVeCVKSTvQ	ASMTL	chrX:1522143-1522193	chrY:1472143-1472193	1	-	38	26	13	10	2	9
uizS5_eieRp9RSuSus	ASMTL	chrX:1522214-1522263	chrY:1472214-1472263	1	-	29	17	13	41	27	15
Zzkrh7v4XVITzYnpZU	ASMTL	chrX:1536914-1536963	chrY:1486914-1486963	1	-	33	21	13	35	21	15
Bc6IklhFV3xJbo79eg	P2RY8	chrX:1581791-1581840	chrY:1531791-1531840	1	-				6	5	2
oVxiXkdfO7iHjhT6_k	DHRSX	chrX:2137782-2137831	chrY:2087782-2087831	1	-	34	24	11	33	20	14
ERl1up1PeyT5Sb3fRs	DHRSX	chrX:2404634-2404683	chrY:2354634-2354683	1	-	35	23	13	24	10	15
f.E1AqoykfoVU91ClY	CD99	chrX:2659031-2659080	chrY:2609031-2609080	1	+	32	21	12	30	19	12
xGhIR55IgflGu.Tnuk	IL9R	chrX:155231087-155231136	chrY:169773-169822	2	+				10	8	3
f1Qp616_CogrQAKG7c	SPRY3	chrX:155011740-155011789	chrY:171456-171505	2	+	33	26	8	27	18	10

Table S4. Demographic and sample information for all samples included in this study.

	All	Male	Female	47,XXY
**Number**	49	31	17	1
**Disease status (SZ:control)**	23:26	12:19	10:07	1:00
**Age (years)**	62 ± 18	59 ± 17	67 ± 20	35
**PMI (hours)**	44 ± 25	35 ± 17	58 ± 31	67
**Prefrontal cortex RIN**	4.5 ± 1.8	4.5 ± 1.9	4.5 ± 1.8	6.9
**Cerebellum RIN**	4.2 ± 2.1	4.3 ± 2.1	4.1 ± 2.4	5.8
**Brain mass (g)**	1410 ± 182	1454 ± 201	1325 ± 92	1417
**Net brain mass (g)**	1251 ± 164	1295 ± 181	1169 ± 82	1306
**Cerebellum mass (g)**	170 ± 24	175 ± 27	160 ± 15	111
**Time since death (years)**	17 ± 4	17 ± 4	17 ± 3	19

PMI, post-mortem interval; RIN, RNA integrity number.

Chromosome	Start	End	Feature	"CER methylation: 47,XXY vs Females"	"CER methylation: 47,XXY vs Males"
chrX	3522732	3522732	cg21030559	0.010586665	0.093454915
chrX	3626829	3626829	cg20578501	0.015029431	0.101712748
chrX	3628424	3628424	cg07501063	-0.006334647	0.093093771
chrX	3630086	3630086	cg17028443	-0.008800574	-0.061436095
chrX	3630407	3630407	cg25601949	-0.010861593	-0.048765034
chrX	3630499	3630499	cg04318657	-0.028156406	-0.049216254
chrX	3630582	3630582	cg16115418	-0.017929618	-0.089688157
chrX	3630619	3630619	cg00658396	-0.011108559	-0.037551192
chrX	3631104	3631104	cg17355083	-0.00523194	-0.01927942
chrX	3631178	3631178	cg08622149	0.283580232	0.298151775
chrX	3631387	3631387	cg13879373	-0.005640252	-0.015870541
chrX	3631575	3631575	cg04065558	0.008986439	-0.018095097
chrX	3631824	3631824	cg18626098	0.053724614	0.04796518
chrX	3632358	3632358	cg09094355	-0.041657251	-0.033335955
chrX	3632422	3632422	cg22364675	-0.012992651	0.146523477
chrX	3632580	3632580	cg06015784	0.05642879	0.033504476
chrX	3633155	3633155	cg17868751	0.022505332	-0.105590322
chrX	6967595	6967595	cg10858432	0.015977141	0.05665366
chrX	7062489	7062489	cg20214316	0.012212855	0.03871568
chrX	7065614	7065614	cg07070940	-0.023674137	-0.021975895
chrX	7065780	7065780	cg11024551	0.003439176	-0.004387669
chrX	7066076	7066076	cg11075227	-0.021934201	-0.020235506
chrX	7066183	7066183	cg03043405	0.000216264	-0.004786073
chrX	7066487	7066487	cg17878951	0.000709578	0.00101674
chrX	7066661	7066661	cg19788004	-0.010595167	-0.037049762
chrX	7066773	7066773	cg26547788	-0.010154786	-0.006337743
chrX	7136807	7136807	cg16232809	-0.017307611	0.005058904
chrX	7136976	7136976	cg10985228	0.060833411	0.185081414
chrX	7137332	7137332	cg00596686	0.002317896	0.011084848
chrX	7137413	7137413	cg15027721	-0.019525065	0.026918055
chrX	7137474	7137474	cg01887803	0.005547856	0.010156686
chrX	7137529	7137529	cg19058005	0.001039714	-0.017544625
chrX	7137913	7137913	cg11640565	0.013376397	0.01394942
chrX	7164772	7164772	cg25700851	0.036089896	0.249759867
chrX	7270088	7270088	cg10073470	-0.000290381	0.02450731
chrX	7867944	7867944	cg24805708	-0.061490321	-0.02046649
chrX	7892382	7892382	cg09972607	0.017573178	0.034687805
chrX	7895031	7895031	cg04659622	-0.034639503	-0.134134392
chrX	7895132	7895132	cg01177664	0.004101595	-0.041983794
chrX	7895379	7895379	cg09659817	-0.009275407	-0.011146429
chrX	7895497	7895497	cg18989977	0.000241747	-0.00750901
chrX	7895503	7895503	cg21604743	0.001227752	-0.017406242
chrX	7895511	7895511	cg00668694	0.019992132	0.000263218
chrX	7895521	7895521	cg22609034	-0.009318067	-0.047764446
chrX	7895531	7895531	cg04640677	-0.020676887	-0.030139199
chrX	7895590	7895590	cg05508067	-0.001859541	-0.017892702
chrX	7895607	7895607	cg01169463	0.009208222	-0.004879003
chrX	7895754	7895754	cg01823238	-0.000284784	0.015378591

chrX	7895861	7895861	cg16832551	-0.002275021	-0.017201252
chrX	7895885	7895885	cg09479579	0.011626337	-0.022370917
chrX	7895900	7895900	cg21864248	0.022208151	-0.004530517
chrX	7896031	7896031	cg03484234	0.049445456	0.023513044
chrX	7896231	7896231	cg18747090	-0.01092066	0.02383391
chrX	7896360	7896360	cg05283871	-0.05452577	-0.003124737
chrX	9430465	9430465	cg00235887	0.056398684	-0.029157837
chrX	9431111	9431111	cg23508627	-0.027484105	-0.099222624
chrX	9431113	9431113	cg17064159	0.019711134	-0.037289203
chrX	9431181	9431181	cg02171705	-0.009292431	-0.024508694
chrX	9431250	9431250	cg04820960	0.001125982	-0.035400576
chrX	9431305	9431305	cg16892191	0.000612293	-0.059479759
chrX	9431326	9431326	cg02263717	0.011534083	-0.014522322
chrX	9432900	9432900	cg10839723	0.030488261	0.033312855
chrX	9432922	9432922	cg22865368	0.001327687	-0.022332258
chrX	9433097	9433097	cg23161024	-0.016025654	-0.005128468
chrX	9433099	9433099	cg13225368	-0.014611222	-0.007326328
chrX	9433103	9433103	cg02810043	-0.046830729	-0.00318294
chrX	9433105	9433105	cg13728104	-0.064074303	0.002196253
chrX	9433262	9433262	cg09524087	-0.017243875	0.005182283
chrX	9433597	9433597	cg14105781	-0.032963321	0.00544158
chrX	9433870	9433870	cg04271131	0.007343581	0.030987431
chrX	9434519	9434519	cg19259802	0.007148202	-0.055961605
chrX	9435372	9435372	cg04414946	-0.068190311	-0.32355177
chrX	9677410	9677410	cg17471720	-0.046585927	0.049823206
chrX	9685813	9685813	cg12035596	0.024403851	0.021811272
chrX	10123962	10123962	cg16888859	0.009954765	0.033438844
chrX	10124333	10124333	cg10775576	-0.014977047	-0.092356107
chrX	10124797	10124797	cg25556752	0.154369057	0.289955476
chrX	10124911	10124911	cg16976876	0.051419451	0.167657426
chrX	10124914	10124914	cg15737470	0.008089692	0.045181863
chrX	10124977	10124977	cg10246296	0.029558245	0.051936229
chrX	10125135	10125135	cg01851385	0.052213923	0.210100927
chrX	10126353	10126353	cg03670355	0.059217029	0.311094333
chrX	10126668	10126668	cg04297907	0.059662013	0.25781352
chrX	10126878	10126878	cg16152676	-0.016685567	0.357169271
chrX	10126881	10126881	cg08333400	-0.010982159	0.084928941
chrX	10128853	10128853	cg11475300	0.007784702	0.133409846

chrX	10128888	10128888	cg00140189	-0.079069308	0.098704083
chrX	10144998	10144998	cg12516234	0.048834476	0.323754165
chrX	10145109	10145109	cg09893016	-0.001110912	0.187034244
chrX	10157676	10157676	cg15374982	-0.026565339	-0.082872198
chrX	10202184	10202184	cg13795116	0.058946479	0.005227162
chrX	11774782	11774782	cg17672846	-0.008483737	-0.022474264
chrX	11775676	11775676	cg25932752	-0.0156861	0.188302989
chrX	11775722	11775722	cg03147775	0.055877442	0.324859782
chrX	11775785	11775785	cg02647401	-0.029486708	0.212517846
chrX	11776121	11776121	cg20775112	0.002091803	0.091025742
chrX	11776256	11776256	cg24195365	-0.00294483	0.247535285
chrX	11776337	11776337	cg15257930	-0.083174212	0.311891248
chrX	11776368	11776368	cg02195366	0.001145041	0.50348477
chrX	11776390	11776390	cg19716713	0.021440056	0.357918992
chrX	11776394	11776394	cg05257947	0.005236687	0.380439776
chrX	11776693	11776693	cg13241003	-0.016369631	0.390183187
chrX	11776853	11776853	cg23484208	0.099966784	0.448416942
chrX	11776935	11776935	cg23590699	-0.032967134	0.323665456
chrX	11777654	11777654	cg16681914	0.115931719	0.400634993
chrX	11777709	11777709	cg02210067	0.105704375	0.207281537
chrX	11777722	11777722	cg05563333	0.006412955	0.22268919
chrX	11777744	11777744	cg07869461	0.067283692	0.308408294
chrX	11777794	11777794	cg01683788	-0.039963906	0.358500686
chrX	11779403	11779403	cg24393665	-0.014485054	0.026111973
chrX	11784864	11784864	cg20205061	0.003126998	0.011612908

chrX	11793466	11793466	cg15695383	-0.04400414	-0.194571835
chrX	13731290	13731290	cg24601489	-0.009957961	-0.001925444
chrX	13733633	13733633	cg13278829	0.018299019	0.050964732
chrX	13735588	13735588	cg24243927	0.032201076	0.073092057
chrX	13749859	13749859	cg19511425	0.025290267	0.055817287
chrX	13751423	13751423	cg05573563	-0.013723397	0.216818101
chrX	13751727	13751727	cg24352688	-0.01662944	0.301320683
chrX	13752442	13752442	cg24714666	-6.99E-06	-0.000720314
chrX	13752670	13752670	cg21308826	-0.027892755	-0.068866516
chrX	13752687	13752687	cg19895492	-0.008037166	-0.02689813
chrX	13752698	13752698	cg04373587	-0.007718206	-0.087577204
chrX	13752760	13752760	cg18634060	-0.004417155	-0.001255842
chrX	13752813	13752813	cg17512860	-0.007218926	0.005475978
chrX	13752880	13752880	cg11104440	0.001064272	-0.045988017
chrX	13752922	13752922	cg22366395	-0.0123998	-0.058042783
chrX	13752933	13752933	cg04096096	0.003840693	-0.022274067
chrX	13752958	13752958	cg23353205	0.000823438	-0.015428019
chrX	13753381	13753381	cg14962776	-0.021380487	-0.03919348
chrX	15755566	15755566	cg00221961	-0.077747517	-0.170261424
chrX	15755989	15755989	cg05059994	0.013015005	0.043670875
chrX	15756052	15756052	cg02283106	0.083007382	0.082075301
chrX	15756059	15756059	cg18391610	0.114847044	0.118286752
chrX	15756062	15756062	cg22702707	0.010775025	-0.064761145
chrX	15756372	15756372	cg00931201	0.044194178	0.007261572
chrX	15756384	15756384	cg18430208	0.0197279	-0.027144283

chrX	15756392	15756392	cg12978205	0.034711575	-0.033462036
chrX	15756407	15756407	cg25954223	-0.008467934	-0.098128829
chrX	15756631	15756631	cg08195028	-0.022772744	-0.066709193
chrX	15756714	15756714	cg25376316	0.0605203	0.078005271
chrX	15756920	15756920	cg05532403	0.056324269	0.008439675
chrX	15758924	15758924	cg07496966	0.023065137	0.102437114
chrX	15768223	15768223	cg07811386	0.017104018	0.023022811
chrX	15804193	15804193	cg12061099	0.004289498	0.011990023
chrX	15807818	15807818	cg01243226	0.021160911	0.022029168
chrX	15808073	15808073	cg22042328	0.013400903	-0.013358993
chrX	15808235	15808235	cg04381324	-0.030663126	-0.120284663
chrX	15808320	15808320	cg13274727	-0.004721908	-0.009527169
chrX	15808390	15808390	cg11353032	-0.012647867	-0.013380121
chrX	15808408	15808408	cg13552866	0.007926966	0.019577197
chrX	15808481	15808481	cg20302203	-0.007823944	-0.012304654
chrX	15808692	15808692	cg19535369	-0.020379418	-0.058516638
chrX	15808900	15808900	cg05012785	-0.000224772	0.014061154
chrX	15809023	15809023	cg03043267	-0.01085227	-0.057449837
chrX	15812307	15812307	cg17790038	0.019369119	0.084617566
chrX	15866587	15866587	cg25363442	0.025007153	0.064241053
chrX	15869341	15869341	cg11635541	0.012512447	0.024779228
chrX	15872216	15872216	cg10321766	-0.015618578	0.023651324
chrX	15872521	15872521	cg20289033	0.000193275	-0.00657732
chrX	15872951	15872951	cg17659886	0.006185417	-0.019146891
chrX	15873151	15873151	cg08313132	0.008015264	-0.029185963

chrX	15873247	15873247	cg01423027	0.005237187	0.027056687
chrX	15873330	15873330	cg18506674	−0.009470875	−0.005777373
chrX	15873337	15873337	cg05106463	−0.00339955	0.01281321
chrX	15873351	15873351	cg05419061	−0.004835829	0.008643409
chrX	15873481	15873481	cg25100404	−0.008951927	−0.01174173
chrX	15873761	15873761	cg18203880	0.017906612	−0.029691494
chrX	15874162	15874162	cg24350752	0.060950498	0.026947479
chrX	16884789	16884789	cg27124447	0.074668738	0.17728948
chrX	16887131	16887131	cg06534187	−0.063664761	0.007534194
chrX	16887664	16887664	cg15464143	−0.083265068	0.083621367
chrX	16887750	16887750	cg21122900	−0.028596162	0.066344998
chrX	16888027	16888027	cg08363715	0.012898814	−0.00795517
chrX	16888211	16888211	cg03811055	−0.023933371	−0.036283522
chrX	16888414	16888414	cg17880859	−0.013688438	−0.018980893
chrX	16888596	16888596	cg07096248	−0.005533045	−0.014895271
chrX	16888606	16888606	cg16978043	0.001950385	0.003156336
chrX	16888622	16888622	cg03048084	−0.010650816	−0.027517512
chrX	16888880	16888880	cg15289846	−0.014057587	0.018067576
chrX	16889152	16889152	cg03171708	0.006087162	0.115180649
chrX	16889173	16889173	cg17878446	0.011372113	0.164126528
chrX	16889247	16889247	cg01947226	−0.062355027	0.196308849
chrX	16889683	16889683	cg14719055	−0.006794872	0.203650281
chrX	20143581	20143581	cg18418138	0.011628713	0.03827937
chrX	20157965	20157965	cg00459767	0.02779082	0.052509295
chrX	20159528	20159528	cg05339472	0.000689762	0.006156365

chrX	20159715	20159715	cg22037115	-0.015431041	-0.035529339
chrX	20159903	20159903	cg12176110	0.013244948	-0.024772999
chrX	20160007	20160007	cg00378717	-0.011949616	-0.004457148
chrX	20160069	20160069	cg14354749	-0.015595748	-0.02872978
chrX	20160086	20160086	cg22264584	-0.011229088	-0.047189708
chrX	20160105	20160105	cg09721174	-0.022728645	-0.079300693
chrX	20160241	20160241	cg19000389	-0.030309541	-0.067618034
chrX	20160243	20160243	cg05257597	-0.03414563	-0.050289041
chrX	20160252	20160252	cg14937106	-0.016520543	-0.006339243
chrX	20161079	20161079	cg11327391	0.002558669	-0.040124745
chrX	21958416	21958416	cg04182378	0.028276877	0.159873182
chrX	21958505	21958505	cg18624866	0.055624643	0.286979551
chrX	21958612	21958612	cg23374711	0.006249477	0.371189496
chrX	21958619	21958619	cg02496423	0.055140128	0.442674349
chrX	21958629	21958629	cg09207137	-0.005028817	0.367121264
chrX	21958631	21958631	cg22417589	-0.023390952	0.219469582
chrX	21958684	21958684	cg02417823	-0.011936783	0.033515176
chrX	21958690	21958690	cg07858069	-0.005849178	0.099897318
chrX	21959520	21959520	cg01445307	-0.00433543	0.38104948
chrX	21959700	21959700	cg03031357	0.123982029	0.383843767
chrX	21960827	21960827	cg09483847	0.041390901	-0.081178512
chrX	21961975	21961975	cg05155058	0.00228745	-0.008303616
chrX	21968128	21968128	cg24980481	-0.037894613	-0.212588538
chrX	22012693	22012693	cg16349029	0.021736742	0.013549252
chrX	24072434	24072434	cg12275687	-0.030983064	-0.051817333

chrX	24072640	24072640	cg09835024	-0.017184082	-0.004468675
chrX	24072811	24072811	cg00060882	0.031461672	0.023709525
chrX	24072865	24072865	cg27345735	0.012010702	-0.017181781
chrX	24072887	24072887	cg12590845	-0.015636493	0.045476489
chrX	24072962	24072962	cg06127902	-0.015552251	-0.059459245
chrX	24073090	24073090	cg16712639	-0.056774595	-0.110342943
chrX	24073134	24073134	cg25034591	0.018063572	0.049217357
chrX	24095462	24095462	cg24530608	-0.021516253	-0.045857795
chrX	24168384	24168384	cg13626059	-0.016277148	-0.012001152
chrX	24168618	24168618	cg15380114	-0.001170812	-0.008226036
chrX	24168784	24168784	cg13653808	0.059454176	0.015463025
chrX	24168810	24168810	cg03159836	-0.014370642	-0.022582572
chrX	24169729	24169729	cg21493591	0.00525402	0.055903388
chrX	24169878	24169878	cg01114138	-0.03318912	0.24668387
chrX	24170765	24170765	cg11001470	0.015173554	0.077222947
chrX	24205107	24205107	cg17546454	0.037186014	0.088207335
chrX	38144773	38144773	cg13780945	0.047104707	0.030370908
chrX	38186399	38186399	cg25933726	0.015666704	0.42798747
chrX	38186472	38186472	cg24741392	0.006035759	0.196815784
chrX	38186700	38186700	cg01121830	-0.052677199	0.206592225
chrX	38186710	38186710	cg00996177	0.016575048	0.375418956
chrX	38186830	38186830	cg02062238	-0.035153376	0.132282971
chrX	38186839	38186839	cg19532714	-0.002328769	0.056539508
chrX	38186842	38186842	cg06944922	-0.022265198	0.067380565
chrX	38186917	38186917	cg01752898	0.033841618	0.194694897

chrX	38186919	38186919	cg23851205	0.027858251	0.07576556
chrX	38187006	38187006	cg07824317	0.190493201	0.358693925
chrX	38187009	38187009	cg03191359	0.059364492	0.481306441
chrX	38187196	38187196	cg01882566	-0.056128296	0.029089466
chrX	40943654	40943654	cg06806679	0.01245639	-0.025625607
chrX	40944327	40944327	cg14099528	-0.015000116	0.002553893
chrX	40944360	40944360	cg05181110	0.013261074	0.025139897
chrX	40944737	40944737	cg18000985	-0.00329562	-0.013999125
chrX	40944772	40944772	cg05196231	-0.002386218	-0.006103588
chrX	40944978	40944978	cg01302641	0.007297508	-0.014142974
chrX	40945574	40945574	cg01501252	0.000414253	0.014950718
chrX	40945750	40945750	cg26039926	-0.009277934	-0.039437906
chrX	40948142	40948142	cg05967389	0.049480888	0.252739834
chrX	40982897	40982897	cg12330929	0.072321996	0.108160617
chrX	41191871	41191871	cg04820200	-0.065129369	0.026473606
chrX	41192164	41192164	cg12689375	-0.018562913	-0.003610675
chrX	41192275	41192275	cg01411845	-0.017273279	-0.016506521
chrX	41192402	41192402	cg17823667	-0.021524669	-0.01598327
chrX	41192529	41192529	cg02892589	-5.56E-05	0.004862216
chrX	41192538	41192538	cg03102848	0.020426757	0.027612138
chrX	41192553	41192553	cg09768249	-0.002813491	0.006310994
chrX	41192872	41192872	cg27042767	-0.008355567	-0.004544745
chrX	41193241	41193241	cg11251991	-0.004688138	-0.028229113
chrX	41193326	41193326	cg16117744	-0.013608049	-0.039414381
chrX	41193409	41193409	cg24751934	-0.001982752	0.002873351

chrX	41193620	41193620	cg06376940	-0.019129934	-0.034245135
chrX	41193886	41193886	cg09523866	-0.002054677	-0.028298793
chrX	41200988	41200988	cg08242820	0.097475003	0.258489457
chrX	41207467	41207467	cg15408180	-0.017367346	0.057217059
chrX	44401762	44401762	cg14084176	0.025367522	-0.00790237
chrX	44402068	44402068	cg07658614	-0.009475329	-0.002135152
chrX	44402332	44402332	cg01573544	-0.010482488	-0.050730512
chrX	44402383	44402383	cg04565250	-0.012065053	0.017646383
chrX	44402448	44402448	cg18511445	0.00707775	-0.033295394
chrX	44403388	44403388	cg02754763	-0.025391981	0.016957189
chrX	44731813	44731813	cg15923127	-0.013592198	-0.006366858
chrX	44732013	44732013	cg20211377	0.002586837	0.001261028
chrX	44732183	44732183	cg03478540	-0.008920318	-0.004980647
chrX	44732348	44732348	cg14384228	-0.002201727	-0.000216821
chrX	44732352	44732352	cg07167981	8.69E-05	-0.005024443
chrX	44732359	44732359	cg27306636	0.006039569	0.000153223
chrX	44732372	44732372	cg11895950	0.011433688	0.004273991
chrX	44732405	44732405	cg17824914	-0.005692716	-0.027105164
chrX	44732417	44732417	cg06877198	-0.032659824	-0.036176547
chrX	44732455	44732455	cg22133177	0.001416302	0.011070357
chrX	44732817	44732817	cg00751785	0.00245306	-0.014798022
chrX	44733206	44733206	cg03267667	-0.008674712	-0.012244537
chrX	44733352	44733352	cg25949649	0.004899269	-0.012365515
chrX	44735560	44735560	cg01888389	-0.033224267	0.266613287
chrX	44970741	44970741	cg18754842	0.001754967	0.127495489

chrX	47049440	47049440	cg08446143	0.028067554	0.000409243
chrX	47049948	47049948	cg27260858	0.011378477	0.132136422
chrX	47049953	47049953	cg27496592	0.039764178	0.241909276
chrX	47050029	47050029	cg25059428	-0.012072573	0.001705226
chrX	47050044	47050044	cg09761980	-0.03046291	0.010342291
chrX	47050093	47050093	cg27564373	0.005583739	0.12395968
chrX	47050139	47050139	cg14251139	0.001976036	0.056705056
chrX	47050149	47050149	cg06895515	0.002832976	0.04409987
chrX	47050152	47050152	cg05153913	0.012975529	0.051856736
chrX	47050154	47050154	cg10717754	0.053496154	0.110412071
chrX	47050208	47050208	cg11235848	-0.003613793	0.042698663
chrX	47050296	47050296	cg22470298	-0.008718978	0.013639179
chrX	47050480	47050480	cg17775283	0.092752232	0.154617354
chrX	47052394	47052394	cg26415216	-0.02151452	-0.054080597
chrX	47052506	47052506	cg09076821	0.005437307	0.010874236
chrX	47052534	47052534	cg14728856	0.07411563	0.082879484
chrX	47053017	47053017	cg19899961	-0.009480423	-0.04467551
chrX	47053031	47053031	cg03149049	-0.000577202	-0.017310662
chrX	47053113	47053113	cg24678093	0.008167729	-0.017703847
chrX	47053154	47053154	cg08341987	-0.007388405	-0.054606562
chrX	47053156	47053156	cg22268449	0.001267247	-0.051887216
chrX	47053161	47053161	cg17699837	0.000277554	-0.028433949
chrX	47053220	47053220	cg06286431	0.025381036	0.012018307
chrX	47053283	47053283	cg07862274	0.016993322	-0.006857125
chrX	47053604	47053604	cg24691157	-0.01018114	-0.028508313

chrX	47053801	47053801	cg17812797	0.005546956	0.013623411
chrX	47054413	47054413	cg02063752	0.005966454	0.385409629
chrX	47057322	47057322	cg19261513	-0.064698192	0.250329954
chrX	47062757	47062757	cg14010367	-0.05444776	0.365468309
chrX	47062794	47062794	cg06242330	-0.016193105	0.120858562
chrX	47063687	47063687	ch.X.772253F	0.176276692	0.326117378
chrX	47063695	47063695	ch.X.772254F	0.028734007	0.193893778
chrX	47064038	47064038	cg12232738	0.022993131	0.13221211
chrX	47064342	47064342	cg18013550	-0.046193246	0.026872586
chrX	47074283	47074283	cg18407858	0.007214483	0.06435972
chrX	47076435	47076435	cg17465884	-0.027356211	0.044453952
chrX	47076559	47076559	cg01203948	-0.016139428	0.279841129
chrX	47077168	47077168	cg10913852	0.035732607	0.056401954
chrX	47077266	47077266	cg13524991	0.024842286	0.035729299
chrX	47077353	47077353	cg12153998	0.067770412	0.114158013
chrX	47077361	47077361	cg06691299	0.062194598	0.228369879
chrX	47077393	47077393	cg16888752	0.058736718	0.104327702
chrX	47077403	47077403	cg05292991	0.060902093	0.141336997
chrX	47077487	47077487	cg13907504	0.075820619	0.121590143
chrX	47077524	47077524	cg19989944	0.021480346	0.02800718
chrX	47077815	47077815	cg14044580	0.044291941	0.09259295
chrX	47077875	47077875	cg17334225	0.009841116	0.017633989
chrX	47077877	47077877	cg11333230	0.112819702	0.139201619
chrX	47077907	47077907	cg13638484	0.045278792	0.170396272
chrX	47077930	47077930	cg23212388	0.034299142	0.09266059

chrX	47077972	47077972	cg19269918	0.02209299	0.08464155
chrX	47077986	47077986	cg22826577	0.049532293	0.140788875
chrX	47078636	47078636	cg17137203	0.01159974	0.134455491
chrX	47079305	47079305	cg03995238	0.008863162	0.300918683
chrX	47079575	47079575	cg23061435	-0.046068657	0.259097081
chrX	47081688	47081688	cg01886810	0.027936375	0.351018658
chrX	47081740	47081740	cg24655012	-0.00460433	0.078850173
chrX	47082076	47082076	cg08710238	-0.070198807	-0.131843088
chrX	47082293	47082293	cg22805594	0.068900991	0.078765153
chrX	47082348	47082348	cg04123347	0.046903812	0.180900649
chrX	47082407	47082407	cg15040559	-0.036071334	0.026403497
chrX	47082510	47082510	cg04317926	0.01563318	0.050740372
chrX	47084401	47084401	cg02424670	0.048345109	0.074196303
chrX	47088999	47088999	cg15604433	-0.023046501	0.016096872
chrX	49046065	49046065	cg15554342	-0.008074091	-0.118513743
chrX	49047791	49047791	cg00581583	-0.077123209	0.249832728
chrX	49047879	49047879	cg22646149	-0.01780586	0.408115217
chrX	49048031	49048031	cg26333397	-0.031644413	0.389163823
chrX	49049363	49049363	cg19786359	-0.122917482	0.142034357
chrX	49050740	49050740	cg25434856	-0.019559137	0.20774654
chrX	49053740	49053740	cg03329583	-0.055595378	0.142702848
chrX	49054690	49054690	cg00768158	-0.067531549	0.205398196
chrX	49056505	49056505	cg11165479	-0.045519429	0.35046392
chrX	49056605	49056605	cg10818284	0.025236209	0.465901403
chrX	49056661	49056661	cg19005062	0.043439459	0.502673326

chrX	49056670	49056670	cg12865398	−0.048089369	0.374783192
chrX	49056686	49056686	cg10983111	0.021356079	0.382956515
chrX	49056688	49056688	cg15199886	−0.028964919	0.413937489
chrX	49056693	49056693	cg12521790	0.001810365	0.387550051
chrX	49056711	49056711	cg15806723	0.083719729	0.331745217
chrX	49056861	49056861	cg20793193	−0.100485443	0.235008641
chrX	49056886	49056886	cg09227616	0.027133657	0.380274261
chrX	49057013	49057013	cg18939543	−0.065244002	0.118041935
chrX	53220515	53220515	cg04751886	−0.010521771	0.095008324
chrX	53247489	53247489	cg10288121	0.055527142	0.100689123
chrX	53251773	53251773	cg22959512	0.01771165	0.340921868
chrX	53253348	53253348	cg03800724	−0.021130624	0.17539931
chrX	53254027	53254027	cg04927657	−0.007396223	−0.01053398
chrX	53254132	53254132	cg09554224	−0.0229333	−0.007418762
chrX	53254643	53254643	cg16824069	−0.001562521	−0.025913312
chrX	53254653	53254653	cg04927982	−0.003164544	−0.041839718
chrX	53254678	53254678	cg19903753	0.002361517	−0.02018764
chrX	53254711	53254711	cg16417926	−0.006022414	−0.019695997
chrX	53254742	53254742	cg27526317	0.000131725	−0.037365092
chrX	53254760	53254760	cg12234996	0.035550587	−0.02799835
chrX	53254818	53254818	cg01859586	0.000746706	−0.030001357
chrX	53255203	53255203	cg24985300	−0.021384607	0.062985022
chrX	53403953	53403953	cg05943755	−0.007145122	0.120915028
chrX	53446266	53446266	cg22629722	−0.021121765	0.116576241
chrX	53449486	53449486	cg07143052	−0.006682579	0.018851405

chrX	53449558	53449558	cg04932149	-0.062075617	0.006606411
chrX	53449561	53449561	cg01404988	-0.010532774	-0.044376416
chrX	53449647	53449647	cg11449070	-0.001117212	0.073795793
chrX	71492485	71492485	cg27359082	-0.063352684	0.007142396
chrX	71494120	71494120	cg20207784	0.024263568	0.173330613
chrX	71496641	71496641	cg06868150	0.00563104	-0.016095249
chrX	71496665	71496665	cg25869832	-0.006410064	-0.010551257
chrX	71496701	71496701	cg01714671	-0.022260966	-0.004218047
chrX	71497035	71497035	cg08859156	-0.009542172	-0.015869074
chrX	71497113	71497113	cg16313903	-0.00441848	0.000724263
chrX	71497221	71497221	cg20882246	-0.006947011	-0.008042409
chrX	71497224	71497224	cg02546818	-0.010892118	0.003044593
chrX	71497301	71497301	cg02575859	0.013092937	-0.013052708
chrX	71497424	71497424	cg16730484	-0.010747992	-0.054874979
chrX	71498442	71498442	cg20551211	0.010633631	0.043822519
chrX	71498597	71498597	cg00008932	0.025030267	-0.120404458
chrX	108913553	108913553	cg18445504	0.00970344	0.061867998
chrX	108976035	108976035	cg15536552	-0.025914446	0.375488373
chrX	108976163	108976163	cg11072201	0.016908251	0.404709163
chrX	108976340	108976340	cg08855111	-0.02848375	0.410191865
chrX	108976619	108976619	cg10721440	-0.058952213	0.279712309
chrX	108976749	108976749	cg09091181	-0.075620066	0.319069652
chrX	108976811	108976811	cg20767561	-0.111466227	0.193484854
chrX	108976825	108976825	cg19635884	-0.109793297	0.237265836
chrX	108976838	108976838	cg26119746	-0.042433841	0.406296649

chrX	108976856	108976856	cg06822229	-0.065565644	0.362135601
chrX	108976893	108976893	cg05441864	-0.096402529	0.298502887
chrX	108977252	108977252	cg14457256	0.069509722	0.20128192
chrX	110923332	110923332	cg13422744	0.036220011	0.0458275
chrX	110923677	110923677	cg23378094	0.001539519	0.01430632
chrX	110924017	110924017	cg24852779	-0.061946375	-0.123094536
chrX	110924347	110924347	cg25834869	-0.027497789	-0.032284756
chrX	110924355	110924355	cg05551025	-0.06496496	0.040423841
chrX	110924389	110924389	cg15612444	-0.011164069	0.119299514
chrX	110924412	110924412	cg12128683	-0.096161419	0.12291852
chrX	110924467	110924467	cg19963797	-0.108686879	0.136677431
chrX	110925015	110925015	cg11152528	-0.115276311	0.038104829
chrX	110925018	110925018	cg16200513	-0.057304518	0.008075677
chrX	110925197	110925197	cg14590681	-0.063304427	0.263112885
chrX	110925365	110925365	cg14233872	-0.111607338	0.01895379
chrX	110928236	110928236	cg26520232	0.032360486	-0.092252474
chrX	110970253	110970253	cg21090723	0.011565672	0.027888286
chrX	139865953	139865953	cg26961103	0.014617808	-0.081796299
chrX	139866026	139866026	cg27074837	0.055288435	0.017230198
chrX	139866080	139866080	cg24922864	0.010739793	0.02476859
chrX	139866322	139866322	cg25937978	-0.001138612	-0.023690048
chrX	139866441	139866441	cg09891468	-0.01077475	-0.062696686
chrX	139866495	139866495	cg08214957	0.035131342	0.011054621
chrX	139866549	139866549	cg19384325	0.033715895	-0.037089416
chrX	139866657	139866657	cg16159925	-0.039466304	-0.188738275

chrX	139868078	139868078	cg02457752	0.014291382	0.00768598
chrY	6777855	6777855	cg02002345	NA	NA
chrY	6778543	6778543	cg27355713	NA	NA
chrY	6778623	6778623	cg04042030	NA	NA
chrY	6778641	6778641	cg02839557	NA	NA
chrY	6778695	6778695	cg01707559	NA	NA
chrY	6778939	6778939	cg15197499	NA	NA
chrY	6780027	6780027	cg09728865	NA	NA
chrY	6781164	6781164	cg01911472	NA	NA
chrY	6783873	6783873	cg27611726	NA	NA
chrY	6891543	6891543	cg08921682	NA	NA
chrY	6954228	6954228	cg15700967	NA	NA
chrY	22736528	22736528	cg01988452	NA	NA
chrY	22736584	22736584	cg13308744	NA	NA
chrY	22736833	22736833	cg10172760	NA	NA
chrY	22737391	22737391	cg10620659	NA	NA
chrY	22737424	22737424	cg15422579	NA	NA
chrY	22737505	22737505	cg15059553	NA	NA
chrY	22737556	22737556	cg01644972	NA	NA
chrY	22737591	22737591	cg02233190	NA	NA
chrY	22737594	22737594	cg26983535	NA	NA
chrY	22737663	22737663	cg11225091	NA	NA
chrY	22737896	22737896	cg03750315	NA	NA
chrY	22737946	22737946	cg02884332	NA	NA
chrY	22737969	22737969	cg08715207	NA	NA
chrY	22738029	22738029	cg08820785	NA	NA
chrY	22741795	22741795	cg00063477	NA	NA
chrY	22754881	22754881	cg01900066	NA	NA

Chromosome	Start	End	Feature	"PFC methylation: 47,XXY vs Females"	PFC methylation: 47XXY vs Males
X	3522732	3522732	cg21030559	0.049742071	0.114909536
X	3626829	3626829	cg20578501	-0.000246937	0.068565536
X	3628424	3628424	cg07501063	-0.131513971	0.010307437
X	3630086	3630086	cg17028443	0.003983032	-0.108599139
X	3630407	3630407	cg25601949	0.010054397	-0.058127463
X	3630499	3630499	cg04318657	-0.031093069	-0.05600216
X	3630582	3630582	cg16115418	-0.026835553	0.012379301
X	3630619	3630619	cg00658396	-0.033324653	-0.048573683
X	3631104	3631104	cg17355083	-0.005358855	-0.019575395
X	3631178	3631178	cg08622149	-0.01099963	-0.004477128
X	3631387	3631387	cg13879373	0.00644418	-0.0054321
X	3631575	3631575	cg04065558	0.008258367	-0.013548906
X	3631824	3631824	cg18626098	-0.025513724	-0.034217097
X	3632358	3632358	cg09094355	-0.047377828	-0.04628847
X	3632422	3632422	cg22364675	-0.150818382	-0.036548776
X	3632580	3632580	cg06015784	0.01264819	-0.105251623
X	3633155	3633155	cg17868751	0.07679536	-0.086053288
X	6967595	6967595	cg10858432	-0.036686913	0.04302073
X	7062489	7062489	cg20214316	0.002835074	0.044367427
X	7065614	7065614	cg07070940	-0.010919665	-0.005540163
X	7065780	7065780	cg11024551	-0.003581237	-0.008246634
X	7066076	7066076	cg11075227	0.00037852	-0.009179756
X	7066183	7066183	cg03043405	0.003704815	-0.00214315
X	7066487	7066487	cg17878951	0.001835826	-0.001317652
X	7066661	7066661	cg19788004	0.008990844	-0.019239977
X	7066773	7066773	cg26547788	-0.009765908	-0.006499953
X	7136807	7136807	cg16232809	-0.009588127	0.003031827
X	7136976	7136976	cg10985228	-0.015036293	0.036670521
X	7137332	7137332	cg00596686	0.018707878	0.037865629
X	7137413	7137413	cg15027721	0.016271635	0.055779122
X	7137474	7137474	cg01887803	0.012675974	0.020571943
X	7137529	7137529	cg19058005	-0.000730381	-0.016068951
X	7137913	7137913	cg11640565	0.026790842	0.006611353
X	7164772	7164772	cg25700851	-0.167476291	0.028692626
X	7270088	7270088	cg10073470	-0.027633762	-0.00356143
X	7867944	7867944	cg24805708	0.012454046	0.062306312
X	7892382	7892382	cg09972607	-0.002240458	0.045084363
X	7895031	7895031	cg04659622	0.057445386	-0.042637068
X	7895132	7895132	cg01177664	-0.011354911	-0.06228163
X	7895379	7895379	cg09659817	0.042209067	0.034327448
X	7895497	7895497	cg18989977	-0.0005072	-0.008881466
X	7895503	7895503	cg21604743	0.011698376	-0.008114891
X	7895511	7895511	cg00668694	-0.025544162	-0.031146218
X	7895521	7895521	cg22609034	-0.007627026	-0.040433123
X	7895531	7895531	cg04640677	0.00417586	0.000349586
X	7895590	7895590	cg05508067	0.003861	-0.009612307
X	7895607	7895607	cg01169463	-0.014103072	-0.020764162
X	7895754	7895754	cg01823238	0.014195287	0.018143046

X	7895861	7895861	cg16832551	−0.034868165	−0.054511357
X	7895885	7895885	cg09479579	−0.013699583	−0.040577012
X	7895900	7895900	cg21864248	−0.029898112	−0.080765116
X	7896031	7896031	cg03484234	0.001278129	−0.035125787
X	7896231	7896231	cg18747090	0.030198378	0.017933699
X	7896360	7896360	cg05283871	−0.01865011	0.053317249
X	9430465	9430465	cg00235887	−0.048134641	−0.110248806
X	9431111	9431111	cg23508627	−0.03733269	−0.098576579
X	9431113	9431113	cg17064159	−0.002193381	−0.062882641
X	9431181	9431181	cg02171705	0.018839113	0.005240307
X	9431250	9431250	cg04820960	0.009136139	−0.034033878
X	9431305	9431305	cg16892191	−0.000575671	−0.063395805
X	9431326	9431326	cg02263717	−0.009544107	−0.026764063
X	9432530	9432530	cg01975591	−0.046958177	−0.147203666
X	9432556	9432556	cg23647143	−0.068754061	−0.146985856
X	9432900	9432900	cg10839723	0.020839801	0.071607209
X	9432922	9432922	cg22865368	−0.015458244	−0.019596689
X	9433097	9433097	cg23161024	−0.024281481	0.000119303
X	9433099	9433099	cg13225368	−0.020500418	0.002476522
X	9433103	9433103	cg02810043	−0.051184904	0.007172262
X	9433105	9433105	cg13728104	−0.032157434	0.058784299
X	9433262	9433262	cg09524087	−0.042969555	0.002072605
X	9433597	9433597	cg14105781	−0.058269413	0.009737926
X	9433870	9433870	cg04271131	−0.024578575	0.024151917
X	9434519	9434519	cg19259802	−0.034418533	−0.067522751
X	9435372	9435372	cg04414946	0.047936437	−0.18847003
X	9584593	9584593	cg15862809	0.05183827	0.14795977
X	9677410	9677410	cg17471720	0.026662279	−0.076105517
X	9685813	9685813	cg12035596	−0.016086404	0.000406464
X	10123962	10123962	cg16888859	0.001761144	0.080202492
X	10124333	10124333	cg10775576	0.029745059	0.158237207
X	10124573	10124573	cg21892973	−0.071197005	0.063844489
X	10124797	10124797	cg25556752	0.004378633	0.129519256
X	10124911	10124911	cg16976876	0.018089055	0.177317462
X	10124914	10124914	cg15737470	−0.050191147	−0.032933903
X	10124977	10124977	cg10246296	0.031675331	0.012216357
X	10125135	10125135	cg01851385	0.012131905	0.106349017
X	10126321	10126321	cg12233790	−0.016677765	0.234905945
X	10126353	10126353	cg03670355	−0.094595447	0.109321986
X	10126668	10126668	cg04297907	−0.006756061	

					0.169829701
X	10126878	10126878	cg16152676	0.026622916	0.204605471
X	10126881	10126881	cg08333400	-0.0443684	-0.039996738
X	10128853	10128853	cg11475300	0.015369785	-0.03322891
X	10128888	10128888	cg00140189	0.03925882	-0.032559951
X	10144998	10144998	cg12516234	0.006496077	0.000733989
X	10145109	10145109	cg09893016	0.015241721	-0.012288816
X	10157676	10157676	cg15374982	-0.004180272	-0.074326791
X	10202184	10202184	cg13795116	-0.065162524	-0.09800685
X	11774782	11774782	cg17672846	0.003330478	0.039821897
X	11775676	11775676	cg25932752	0.036811943	0.228004902
X	11775722	11775722	cg03147775	0.07098907	0.318645199
X	11775785	11775785	cg02647401	-0.001015227	0.233395385
X	11776121	11776121	cg20775112	0.027548015	0.113390492
X	11776256	11776256	cg24195365	0.004079442	0.248230639
X	11776337	11776337	cg15257930	-0.009192525	0.406455351
X	11776368	11776368	cg02195366	-0.024896649	0.481027297
X	11776390	11776390	cg19716713	0.021953258	0.371201364
X	11776394	11776394	cg05257947	0.009159136	0.394326351
X	11776693	11776693	cg13241003	0.055883399	0.45017044
X	11776853	11776853	cg23484208	0.048202582	0.384149668
X	11776935	11776935	cg23590699	0.019398398	0.380432252
X	11777654	11777654	cg16681914	0.001405771	0.32226852
X	11777709	11777709	cg02210067	0.003065428	0.098729359
X	11777722	11777722	cg05563333	0.034747368	0.273539751
X	11777744	11777744	cg07869461	0.003793389	

					0.253307365
X	11777794	11777794	cg01683788	0.077079609	0.475312341
X	11779403	11779403	cg24393665	0.031573101	0.121246308
X	11784864	11784864	cg20205061	-0.081087409	-0.001334944
X	11793466	11793466	cg15695383	0.021440845	-0.016586558
X	13731290	13731290	cg24601489	0.012252718	0.034378524
X	13735588	13735588	cg24243927	-0.00732413	0.060802955
X	13749859	13749859	cg19511425	-0.009929279	0.031506551
X	13751423	13751423	cg05573563	0.038935646	0.19026048
X	13751727	13751727	cg24352688	-0.03309377	0.191941978
X	13752442	13752442	cg24714666	-0.001799156	0.005376199
X	13752670	13752670	cg21308826	0.016924226	-0.009273946
X	13752687	13752687	cg19895492	-0.000985067	-0.017276945
X	13752698	13752698	cg04373587	0.008147452	-0.032608146
X	13752760	13752760	cg18634060	-0.013473908	6.78E-05
X	13752813	13752813	cg17512860	-0.015825218	0.016350803
X	13752880	13752880	cg11104440	-0.043218577	-0.038120751
X	13752922	13752922	cg22366395	0.001603153	-0.033738902
X	13752933	13752933	cg04096096	-0.02558782	-0.041357666
X	13752958	13752958	cg23353205	-0.002662237	-0.017654514
X	13753119	13753119	cg05598468	0.057796849	0.009294808
X	13753381	13753381	cg14962776	-0.003334085	-0.002889376
X	15755566	15755566	cg00221961	0.012626881	-0.006266194
X	15755989	15755989	cg05059994	-0.022291778	-0.03011293
X	15756052	15756052	cg02283106	-0.018370724	0.013247367
X	15756059	15756059	cg18391610	-0.040969405	

					-0.022468259
X	15756062	15756062	cg22702707	-0.008885392	-0.076182955
X	15756312	15756312	cg12386568	0.028145556	-0.082217646
X	15756372	15756372	cg00931201	-0.03336881	-0.042785388
X	15756384	15756384	cg18430208	0.009173419	-0.009213852
X	15756392	15756392	cg12978205	0.014930888	-0.057674589
X	15756407	15756407	cg25954223	-0.0049614	-0.084601968
X	15756631	15756631	cg08195028	-0.005951296	-0.015333993
X	15756714	15756714	cg25376316	0.002138681	0.012567561
X	15756920	15756920	cg05532403	0.058994842	1.55E-05
X	15758924	15758924	cg07496966	0.007423978	0.093115241
X	15768223	15768223	cg07811386	-0.001355677	0.071109535
X	15807818	15807818	cg01243226	0.024490872	0.029914722
X	15808073	15808073	cg22042328	-0.002038251	0.006412891
X	15808235	15808235	cg04381324	3.57E-05	-0.080549667
X	15808320	15808320	cg13274727	0.000876518	0.000136117
X	15808390	15808390	cg11353032	-0.01823529	-0.010335869
X	15808408	15808408	cg13552866	-0.022221928	-0.020756664
X	15808481	15808481	cg20302203	0.011054778	0.00949064
X	15808692	15808692	cg19535369	-0.00750142	-0.044542485
X	15808900	15808900	cg05012785	0.010450939	0.01590148
X	15809023	15809023	cg03043267	-0.0125341	-0.056352479
X	15812307	15812307	cg17790038	0.069044787	0.080881623
X	15866587	15866587	cg25363442	0.034164609	0.105764277
X	15869341	15869341	cg11635541	0.033777438	0.04409043
X	15871902	15871902	cg02167358	0.020388614	

					-0.057619207
X	15872216	15872216	cg10321766	0.016578275	0.048001236
X	15872521	15872521	cg20289033	-0.00805222	-0.015315937
X	15872951	15872951	cg17659886	-0.015616477	-0.03414885
X	15873151	15873151	cg08313132	-0.001692431	-0.035610177
X	15873247	15873247	cg01423027	-0.007262317	0.025196507
X	15873330	15873330	cg18506674	-0.02259467	0.015352562
X	15873337	15873337	cg05106463	-0.012521828	0.034088337
X	15873351	15873351	cg05419061	-0.013521429	0.004923705
X	15873481	15873481	cg25100404	-0.016204547	-0.013262985
X	15873761	15873761	cg18203880	0.013433329	-0.076519954
X	15874162	15874162	cg24350752	-0.003260464	-0.099054965
X	16863146	16863146	cg06231015	0.027786843	0.12909862
X	16884789	16884789	cg27124447	0.138708557	0.277612541
X	16887131	16887131	cg06534187	-0.047037876	0.056293025
X	16887664	16887664	cg15464143	0.003915445	0.223369937
X	16887750	16887750	cg21122900	-0.012696251	0.109249868
X	16888027	16888027	cg08363715	-0.017330865	-0.024724599
X	16888211	16888211	cg03811055	-0.035938279	-0.007314257
X	16888414	16888414	cg17880859	-0.030347243	-0.022691816
X	16888596	16888596	cg07096248	-0.018592739	-0.012592446
X	16888606	16888606	cg16978043	-0.014014601	0.015667449
X	16888622	16888622	cg03048084	-0.009124813	0.008368468
X	16888880	16888880	cg15289846	-0.031121432	0.032496919
X	16889152	16889152	cg03171708	-0.07316932	0.122782153
X	16889173	16889173	cg17878446	-0.062018605	

					0.226534331
X	16889247	16889247	cg01947226	-0.011548315	0.280100668
X	16889683	16889683	cg14719055	0.050425893	0.196139988
X	20143581	20143581	cg18418138	0.016988239	0.081901508
X	20157965	20157965	cg00459767	0.032717824	0.087855226
X	20159528	20159528	cg05339472	-0.012045869	-0.003322697
X	20159715	20159715	cg22037115	-0.024801759	-0.03448417
X	20159903	20159903	cg12176110	-0.023707448	-0.038507203
X	20160007	20160007	cg00378717	-0.025217228	-0.010009159
X	20160069	20160069	cg14354749	-0.025134808	-0.015878868
X	20160086	20160086	cg22264584	-0.009970683	-0.026506675
X	20160105	20160105	cg09721174	0.006208339	-0.034323091
X	20160241	20160241	cg19000389	-0.041738563	-0.058719478
X	20160243	20160243	cg05257597	-0.012964905	-0.028272855
X	20160252	20160252	cg14937106	-0.023167865	0.018348309
X	20161079	20161079	cg11327391	-0.00841149	-0.027597011
X	21958416	21958416	cg04182378	0.010055236	0.140849788
X	21958505	21958505	cg18624866	-0.000740156	0.240864603
X	21958612	21958612	cg23374711	0.021617781	0.39988632
X	21958619	21958619	cg02496423	-0.020726182	0.388788742
X	21958629	21958629	cg09207137	-0.005653935	0.369348731
X	21958631	21958631	cg22417589	-0.011171689	0.267282871
X	21958684	21958684	cg02417823	-0.029641482	0.033209693
X	21958690	21958690	cg07858069	-0.002779305	0.117366051
X	21959520	21959520	cg01445307	0.065104357	0.435234237
X	21959700	21959700	cg03031357	0.040743863	

					0.316790698
X	21960400	21960400	cg23161359	0.012036915	0.100772309
X	21960827	21960827	cg09483847	−0.015018885	−0.138014673
X	21961975	21961975	cg05155058	−0.007921804	−0.138628516
X	21968128	21968128	cg24980481	0.026803768	−0.168840986
X	22012693	22012693	cg16349029	0.015770652	0.010031199
X	24072434	24072434	cg12275687	0.058972536	0.027251151
X	24072640	24072640	cg09835024	0.018804934	0.037490283
X	24072811	24072811	cg00060882	−0.020507205	−0.033120542
X	24072865	24072865	cg27345735	0.023605811	0.023182987
X	24072887	24072887	cg12590845	−0.013277652	0.042067444
X	24072962	24072962	cg06127902	−0.004687685	−0.018359633
X	24073090	24073090	cg16712639	0.032963916	0.017041645
X	24073134	24073134	cg25034591	−0.011219971	0.023939069
X	24095462	24095462	cg24530608	0.000328886	0.026671144
X	24168384	24168384	cg13626059	0.002238038	−0.005215094
X	24168618	24168618	cg15380114	−0.001702263	−0.009835051
X	24168784	24168784	cg13653808	0.050827096	0.004329359
X	24168810	24168810	cg03159836	−0.00255092	−0.011675687
X	24169729	24169729	cg21493591	0.004769523	0.049920384
X	24169878	24169878	cg01114138	0.008797226	0.120650173
X	24170765	24170765	cg11001470	−0.035296503	0.019157071
X	38186399	38186399	cg25933726	−0.001078461	0.380466908
X	38186472	38186472	cg24741392	−0.011694707	0.21488822
X	38186700	38186700	cg01121830	−0.019376019	0.254926042
X	38186710	38186710	cg00996177	−0.018368754	

					0.345406248
X	38186830	38186830	cg02062238	−0.037119966	0.160383953
X	38186839	38186839	cg19532714	0.014240734	0.099249396
X	38186842	38186842	cg06944922	0.057506959	0.178234115
X	38186917	38186917	cg01752898	0.02764085	0.210950627
X	38186919	38186919	cg23851205	−0.015438202	0.05203341
X	38187006	38187006	cg07824317	−0.032094591	0.130501778
X	38187009	38187009	cg03191359	−0.015946818	0.367985818
X	38187196	38187196	cg01882566	0.069865711	0.119236635
X	40943654	40943654	cg06806679	0.012390792	−0.034457043
X	40944327	40944327	cg14099528	−0.01537303	−0.00990269
X	40944360	40944360	cg05181110	−0.01222415	−0.010671138
X	40944737	40944737	cg18000985	−0.001148769	−0.016702148
X	40944772	40944772	cg05196231	4.03E−05	−0.008640214
X	40944978	40944978	cg01302641	−0.007560518	−0.024172202
X	40945574	40945574	cg01501252	−0.022255622	−0.016208323
X	40945750	40945750	cg26039926	−0.005475744	−0.037517416
X	40948142	40948142	cg05967389	0.013736208	0.253144428
X	40982897	40982897	cg12330929	0.001823035	0.044303012
X	41191871	41191871	cg04820200	−0.035041811	0.000843936
X	41192164	41192164	cg12689375	−0.028797904	−0.044830101
X	41192275	41192275	cg01411845	−0.015214555	−0.009263691
X	41192402	41192402	cg17823667	−0.012918082	−0.006229891
X	41192529	41192529	cg02892589	−0.005632908	−0.002657076
X	41192538	41192538	cg03102848	−0.004911551	0.001433174
X	41192553	41192553	cg09768249	−0.019255144	

					-0.010964663
X	41192872	41192872	cg27042767	-0.006557762	-0.004821667
X	41193241	41193241	cg11251991	-0.006964746	-0.026636865
X	41193326	41193326	cg16117744	0.002215108	-0.025563515
X	41193409	41193409	cg24751934	-0.007494594	-0.006316765
X	41193620	41193620	cg06376940	-0.000560275	-0.011555118
X	41193886	41193886	cg09523866	0.00229768	-0.029582187
X	41200988	41200988	cg08242820	0.008619388	0.167637637
X	41207467	41207467	cg15408180	-0.005983125	0.058977049
X	44401762	44401762	cg14084176	-0.008847396	-0.016648125
X	44402068	44402068	cg07658614	-0.018409165	-8.81E-05
X	44402332	44402332	cg01573544	-0.004363255	-0.037559797
X	44402383	44402383	cg04565250	-0.031964099	0.035348777
X	44402448	44402448	cg18511445	0.011780298	0.00047506
X	44403388	44403388	cg02754763	0.005568009	0.075683897
X	44731813	44731813	cg15923127	0.009261969	0.007625639
X	44732013	44732013	cg20211377	-0.00929508	-0.007896037
X	44732183	44732183	cg03478540	-0.01384752	-0.016180039
X	44732348	44732348	cg14384228	-0.029819843	-0.027511999
X	44732352	44732352	cg07167981	-0.010588599	-0.017366374
X	44732359	44732359	cg27306636	-0.007578142	-0.012159629
X	44732372	44732372	cg11895950	0.002982898	-0.003659632
X	44732405	44732405	cg17824914	0.000641937	-0.010787079
X	44732417	44732417	cg06877198	0.011875593	0.006621324
X	44732455	44732455	cg22133177	-0.005531018	0.003506962
X	44732817	44732817	cg00751785	0.0107215	

					−0.000982041
X	44733206	44733206	cg03267667	−0.00026973	−0.000999406
X	44733352	44733352	cg25949649	−0.001888949	−0.019474944
X	44735560	44735560	cg01888389	−0.02472006	0.172652198
X	44970741	44970741	cg18754842	0.028052291	0.096336618
X	47049440	47049440	cg08446143	0.017504257	−0.011878515
X	47049948	47049948	cg27260858	−0.042547655	0.093498689
X	47049953	47049953	cg27496592	−0.01633694	0.1998485
X	47050029	47050029	cg25059428	−0.023842282	0.021484057
X	47050044	47050044	cg09761980	−0.038040058	0.039688121
X	47050093	47050093	cg27564373	0.00223273	0.155825565
X	47050139	47050139	cg14251139	−0.031022998	0.073948523
X	47050149	47050149	cg06895515	−0.021847573	0.045199403
X	47050152	47050152	cg05153913	0.004972944	0.04495135
X	47050154	47050154	cg10717754	0.003245567	0.065791101
X	47050208	47050208	cg11235848	−0.043664539	0.045591746
X	47050296	47050296	cg22470298	−0.01224839	0.053674244
X	47050480	47050480	cg17775283	−0.047000927	0.053465748
X	47052394	47052394	cg26415216	−0.02944304	−0.052081505
X	47052506	47052506	cg09076821	0.006506584	0.048123452
X	47052534	47052534	cg14728856	−0.012239337	−0.063118027
X	47053017	47053017	cg19899961	−0.009623822	−0.041889139
X	47053031	47053031	cg03149049	−0.010015003	−0.032204011
X	47053113	47053113	cg24678093	0.001326449	−0.021847751
X	47053154	47053154	cg08341987	0.012117842	−0.040134274
X	47053156	47053156	cg22268449	0.011074018	

	−0.066454733			
X	47053161	47053161	cg17699837	0.007625204
	−0.016164312			
X	47053220	47053220	cg06286431	−0.021974471
	−0.036416337			
X	47053283	47053283	cg07862274	−0.011877164
	−0.037797728			
X	47053604	47053604	cg24691157	−0.004292734
	−0.010624812			
X	47053801	47053801	cg17812797	−0.012893887
	0.001147027			
X	47054413	47054413	cg02063752	0.051928544
	0.288665655			
X	47057322	47057322	cg19261513	0.022513457
	0.220634216			
X	47062757	47062757	cg14010367	0.035945581
	0.180839925			
X	47062794	47062794	cg06242330	−0.012987565
	0.014408098			
X	47063687	47063687	ch.X.772253F	0.017266961
	0.135067309			
X	47063695	47063695	ch.X.772254F	0.032446878
	0.186656777			
X	47064038	47064038	cg12232738	−0.021400084
	0.010405872			
X	47064342	47064342	cg18013550	−0.012108261
	−0.0028427			
X	47074283	47074283	cg18407858	−0.016507753
	0.007617786			
X	47076435	47076435	cg17465884	−0.020727622
	0.046620924			
X	47076559	47076559	cg01203948	0.030301406
	0.281147488			
X	47077168	47077168	cg10913852	0.004795078
	0.040709009			
X	47077266	47077266	cg13524991	−0.014208924
	0.020716501			
X	47077353	47077353	cg12153998	0.013110876
	0.106496374			
X	47077361	47077361	cg06691299	0.017822662
	0.238449			
X	47077393	47077393	cg16888752	−0.026852975
	0.07303993			
X	47077403	47077403	cg05292991	−0.074515332
	0.044486196			
X	47077487	47077487	cg13907504	0.021771929
	0.083262768			
X	47077524	47077524	cg19989944	0.009442568
	0.014188003			
X	47077815	47077815	cg14044580	−0.004777733

					0.03939677
X	47077875	47077875	cg17334225	0.002887472	0.030923384
X	47077877	47077877	cg11333230	0.012913237	0.083306372
X	47077907	47077907	cg13638484	-0.0162429	0.104151734
X	47077930	47077930	cg23212388	-0.021535851	0.057106031
X	47077972	47077972	cg19269918	0.002861766	0.069573456
X	47077986	47077986	cg22826577	-0.049035306	0.036849295
X	47078636	47078636	cg17137203	-0.000172007	0.181982884
X	47079305	47079305	cg03995238	0.0398182	0.226088871
X	47079575	47079575	cg23061435	0.035616918	0.241797212
X	47081688	47081688	cg01886810	0.011320857	0.027299876
X	47081740	47081740	cg24655012	-0.004357468	-0.029119697
X	47082076	47082076	cg08710238	0.025737393	-0.015570263
X	47082293	47082293	cg22805594	0.048178888	0.034807916
X	47082348	47082348	cg04123347	0.032506077	0.11929472
X	47082407	47082407	cg15040559	0.012850199	0.03828259
X	47082510	47082510	cg04317926	-0.005296263	0.002001519
X	47084401	47084401	cg02424670	-0.002383487	0.034307686
X	47088999	47088999	cg15604433	0.04584226	0.054567131
X	49046065	49046065	cg15554342	-0.05386522	-0.124309584
X	49047791	49047791	cg00581583	0.005456936	0.327606694
X	49047879	49047879	cg22646149	-0.023927746	0.386706194
X	49048031	49048031	cg26333397	0.024649145	0.441030575
X	49049363	49049363	cg19786359	-0.014632105	0.066278344
X	49050740	49050740	cg25434856	0.030289466	0.047690841
X	49053740	49053740	cg03329583	0.046610412	

					0.024719491
X	49054690	49054690	cg00768158	0.057743025	0.176774207
X	49056505	49056505	cg11165479	0.053376119	0.434944664
X	49056605	49056605	cg10818284	−0.016820875	0.450880128
X	49056661	49056661	cg19005062	−0.028864147	0.419100921
X	49056670	49056670	cg12865398	−0.009458163	0.412454058
X	49056686	49056686	cg10983111	−0.012077575	0.359591014
X	49056688	49056688	cg15199886	−0.009802202	0.413583689
X	49056693	49056693	cg12521790	−0.037544245	0.354911216
X	49056711	49056711	cg15806723	0.03557027	0.307361443
X	49056861	49056861	cg20793193	−0.037229805	0.300010067
X	49056886	49056886	cg09227616	−0.010102529	0.370550975
X	49057013	49057013	cg18939543	−0.031845404	0.159744625
X	53220515	53220515	cg04751886	−0.02709403	0.078314722
X	53247489	53247489	cg10288121	0.004224746	0.036678906
X	53251773	53251773	cg22959512	0.012365322	0.19291722
X	53253348	53253348	cg03800724	0.013163334	0.19617335
X	53254027	53254027	cg04927657	−0.010285287	0.00037648
X	53254132	53254132	cg09554224	−0.013421097	−0.003740231
X	53254643	53254643	cg16824069	0.000151386	−0.023029995
X	53254653	53254653	cg04927982	0.005394565	−0.032442921
X	53254678	53254678	cg19903753	−0.005692761	−0.021155467
X	53254711	53254711	cg16417926	−0.000261756	−0.010588519
X	53254742	53254742	cg27526317	0.003898219	−0.017344882
X	53254760	53254760	cg12234996	−0.009350733	−0.054879136
X	53254818	53254818	cg01859586	−0.001732778	

					-0.024437222
X	53255203	53255203	cg24985300	0.056319442	0.148490813
X	53403953	53403953	cg05943755	0.029432379	0.025199957
X	53446266	53446266	cg22629722	0.022452158	0.099592753
X	53449486	53449486	cg07143052	-0.007761933	0.04973191
X	53449558	53449558	cg04932149	-0.025578696	0.041505244
X	53449561	53449561	cg01404988	0.005378933	-0.007816468
X	53449647	53449647	cg11449070	-0.071167104	0.09678575
X	71492485	71492485	cg27359082	-0.018430591	0.056526998
X	71494120	71494120	cg20207784	-0.019704051	0.105798121
X	71496641	71496641	cg06868150	-0.001664438	0.01312248
X	71496665	71496665	cg25869832	-0.032821785	-0.004332275
X	71496701	71496701	cg01714671	0.006954715	0.114732193
X	71497035	71497035	cg08859156	-0.004336423	0.010143459
X	71497113	71497113	cg16313903	0.012440223	0.04893103
X	71497221	71497221	cg20882246	-0.021891543	0.001829744
X	71497224	71497224	cg02546818	-0.00460789	0.042189791
X	71497301	71497301	cg02575859	-0.029313751	-0.036820146
X	71497424	71497424	cg16730484	0.014465091	0.000994325
X	71498442	71498442	cg20551211	0.015950133	0.042739699
X	71498597	71498597	cg00008932	-0.020569786	-0.197314557
X	108913553	108913553	cg18445504	-0.022577235	0.050525952
X	108976035	108976035	cg15536552	-0.021953824	0.425887061
X	108976163	108976163	cg11072201	0.002158096	0.411546067
X	108976340	108976340	cg08855111	-0.007123919	0.437946699
X	108976619	108976619	cg10721440	-0.108225827	

					0.247267304
X	108976749	108976749	cg09091181	-0.004093519	0.398621039
X	108976811	108976811	cg20767561	0.040263437	0.339995156
X	108976825	108976825	cg19635884	-0.042163851	0.296205877
X	108976838	108976838	cg26119746	0.007122102	0.437170501
X	108976856	108976856	cg06822229	0.043188407	0.476056874
X	108976893	108976893	cg05441864	-0.053057739	0.299577108
X	108977252	108977252	cg14457256	0.00510279	0.089612946
X	110923332	110923332	cg13422744	-0.093041401	-0.059762585
X	110923677	110923677	cg23378094	0.002635985	0.022485271
X	110924017	110924017	cg24852779	0.003938183	-0.026632273
X	110924347	110924347	cg25834869	-0.028085651	-0.020321631
X	110924355	110924355	cg05551025	-0.068099578	0.074290976
X	110924389	110924389	cg15612444	-0.053600734	0.104289578
X	110924412	110924412	cg12128683	-0.085851113	0.183221338
X	110924467	110924467	cg19963797	-0.046064254	0.226805518
X	110925015	110925015	cg11152528	-0.056238005	0.133937406
X	110925018	110925018	cg16200513	-0.066897378	0.030145222
X	110925197	110925197	cg14590681	-0.109326149	0.21821986
X	110925365	110925365	cg14233872	-0.080987951	0.050330791
X	110928236	110928236	cg26520232	0.048688979	0.061423658
X	110970253	110970253	cg21090723	-0.026520676	0.035930095
X	139865953	139865953	cg26961103	0.03529017	-0.006687245
X	139866026	139866026	cg27074837	-0.020107035	-0.051547491
X	139866080	139866080	cg24922864	0.006334241	0.035446906
X	139866322	139866322	cg25937978	0.018057771	

					−0.021774255
X	139866441	139866441	cg09891468	−0.032547671	−0.048443958
X	139866495	139866495	cg08214957	0.008331709	0.001842188
X	139866549	139866549	cg19384325	0.022710231	−0.065759188
X	139866657	139866657	cg16159925	−0.044241179	−0.169599575
X	139868078	139868078	cg02457752	0.007669037	0.051419023
Y	6777855	6777855	cg02002345	NA	−0.033679283
Y	6778543	6778543	cg27355713	NA	−0.003163807
Y	6778623	6778623	cg04042030	NA	−0.005570621
Y	6778641	6778641	cg02839557	NA	−0.008247955
Y	6778695	6778695	cg01707559	NA	−0.002872333
Y	6778939	6778939	cg15197499	NA	−0.016702666
Y	6780027	6780027	cg09728865	NA	0.040196479
Y	6781164	6781164	cg01911472	NA	−0.005950049
Y	6783873	6783873	cg27611726	NA	−0.011150979
Y	6891543	6891543	cg08921682	NA	−0.041590235
Y	6954228	6954228	cg15700967	NA	−0.022583199
Y	22736528	22736528	cg01988452	NA	0.048213355
Y	22736584	22736584	cg13308744	NA	0.069516814
Y	22736833	22736833	cg10172760	NA	0.119563565
Y	22737391	22737391	cg10620659	NA	0.009707997
Y	22737424	22737424	cg15422579	NA	−0.012459799
Y	22737505	22737505	cg15059553	NA	−0.056071915
Y	22737556	22737556	cg01644972	NA	−0.034170079
Y	22737591	22737591	cg02233190	NA	−0.016781928
Y	22737594	22737594	cg26983535	NA	−0.00965339
Y	22737663	22737663	cg11225091	NA	−0.005979725
Y	22737896	22737896	cg03750315	NA	−0.035291141
Y	22737946	22737946	cg02884332	NA	−0.007295951
Y	22737969	22737969	cg08715207	NA	0.030065922
Y	22738029	22738029	cg08820785	NA	−0.050272389

Y	22741795	22741795	cg00063477	NA	0.040698013
Y	22754881	22754881	cg01900066	NA	0.081772151

Chromosome	Start	End	Feature	"CER expression: 47,XXY vs Females"	"CER expression: 47,XXY vs Males"
chrX	219773	219822	PLCXD1	0.407470914	0.096550501
chrX	221456	221505	GTPBP6	-0.682366356	-0.916689491
chrX	299374	299423	PPP2R3B	0.101780712	0.014006094
chrX	306290	306339	PPP2R3B	0.104278338	0.080901114
chrX	1327710	1327759	CRLF2	-0.034681036	-0.034969633
chrX	1428309	1428358	CSF2RA	0.006653826	-0.007609103
chrX	1428310	1428359	CSF2RA	0.049272286	0.025780317
chrX	1428704	1428753	CSF2RA	-0.827269412	-0.632183906
chrX	1501338	1501387	IL3RA	-0.02917637	-0.016317556
chrX	1505329	1505378	SLC25A6	0.812418271	0.392734961
chrX	1522143	1522193	ASMTL	-0.023620604	-0.401447976
chrX	1522214	1522263	ASMTL	0.640160481	0.277928608
chrX	1536914	1536963	ASMTL	0.386553073	0.244135565
chrX	1581791	1581840	P2RY8	-0.033614195	-0.04331058
chrX	2137782	2137831	DHRSX	0.111781021	0.043564462
chrX	2404634	2404683	DHRSX	0.319536273	-0.010756246
chrX	2659031	2659080	CD99	0.150147449	0.065301529
chrX	3522660	3522709	PRKX	-0.219932093	-0.173137274
chrX	3522945	3522994	PRKX	-0.128113349	-0.036810045
chrX	6967201	6967250	HDHD1	-0.091280046	0.048290458
chrX	7272357	7272406	STS	0.04855497	0.085199975
chrX	7867185	7867234	PNPLA4	0.188171227	0.159672062
chrX	9687610	9687659	TBL1X	-0.354740249	-0.297585181
chrX	10203332	10203381	CLCN4	-0.007910923	0.01560711
chrX	11778975	11779024	MSL3	-0.323972782	-0.302842848
chrX	11783794	11783843	MSL3	-0.040885171	-0.048153663
chrX	11785722	11785771	MSL3	-0.001644013	-0.037236118
chrX	11785862	11785911	MSL3	-0.04966711	-0.032552108
chrX	13730737	13730786	TRAPPC2	-0.032381464	0.063427078
chrX	13731262	13731311	TRAPPC2	-0.054475962	-0.044306957
chrX	13752206	13752255	TRAPPC2	-0.020164638	-0.01091678
chrX	15802415	15802464	CA5B	0.043832641	0.052763566
chrX	15841166	15841215	ZRSR2	-0.22800547	-0.180624291
chrX	15844225	15844274	AP1S2	0.591786286	0.478183018
chrX	15844477	15844526	AP1S2	0.627708218	0.524356115
chrX	16863144	16863193	RBBP7	0.333562651	

0.179868059
chrX	17897216	17897231
CRLF2	-0.024747262	-0.017823853
chrX	20142985	20143034	EIF1AX	0.762775937
0.807753674
chrX	20148703	20148725	EIF1AX	0.665954293
0.696282857
chrX	20150300	20150326	EIF1AX	0.665954293
0.696282857
chrX	22010730	22010779	SMS	0.797606437
0.660439008
chrX	22010739	22010788	SMS	0.459407395
0.312665772
chrX	24095795	24095844	EIF2S3	0.328556534
0.396209051
chrX	24231696	24231745	ZFX	0.02855762
0.242587375
chrX	38128919	38128968	RPGR	0.022386893
0.046504489
chrX	38144030	38144079	RPGR
0.02256286	-0.013344917
chrX	38156698	38156705
RPGR	-0.108901786	-0.072568996
chrX	38158209	38158250
RPGR	-0.108901786	-0.072568996
chrX	41092817	41092866	USP9X	0.402648948
0.495533541
chrX	41095608	41095657	USP9X	0.338769733
0.419791299
chrX	41208966	41209015	DDX3X	0.203024248
0.564243065
chrX	44383336	44383385	FUNDC1	0.08744631
0.015427183
chrX	44970808	44970857	KDM6A	-0.047292548
0.108220972
chrX	47074273	47074322	UBA1	0.026101693
0.04207599
chrX	49044434	49044483	SYP	0.821452055
0.570653938
chrX	53401356	53401405	SMC1A	0.181529323
0.067964958
chrX	71493707	71493756
RPS4X	-0.047086808	-0.029085322
chrX	71495532	71495574	RPS4X	-0.07627071
0.12916278
chrX	71496007	71496013	RPS4X	-0.07627071
0.12916278
chrX	108884977	108885026	ACSL4	0.470177281
0.708973315
chrX	108911443	108911492	ACSL4	0.302044137

0.263911301
chrX 108926784 108926833
ACSL4 -0.083882215 -0.06749
chrX 110928303 110928331
ALG13 -0.033661694 -0.022618383
chrX 110931115 110931135
ALG13 -0.033661694 -0.022618383
chrX 110931892 110931941 ALG13 0.060953756
0.115655255
chrX 155011740 155011789 SPRY3 0.014183914
0.010475217
chrX 155172999 155173048 VAMP7 0.454966909
0.460256868
chrX 155231087 155231136 IL9R
0.010313296 -0.02177692
chrY 169773 169822 PLCXD1 0.407470914 0.096550501
chrY 171456 171505 GTPBP6 -0.682366356 -0.916689491
chrY 249374 249423 PPP2R3B 0.101780712 0.014006094
chrY 256290 256339 PPP2R3B 0.104278338 0.080901114
chrY 1277710 1277759 CRLF2 -0.034681036 -0.034969633
chrY 1378309 1378358 CSF2RA 0.006653826 -0.007609103
chrY 1378310 1378359 CSF2RA 0.049272286 0.025780317
chrY 1378704 1378753 CSF2RA -0.827269412 -0.632183906
chrY 1451338 1451387 IL3RA -0.02917637 -0.016317556
chrY 1455329 1455378 SLC25A6 0.812418271 0.392734961
chrY 1472143 1472193 ASMTL -0.023620604 -0.401447976
chrY 1472214 1472263 ASMTL 0.640160481 0.277928608
chrY 1486914 1486963 ASMTL 0.386553073 0.244135565
chrY 1531791 1531840 P2RY8 -0.033614195 -0.04331058
chrY 2087782 2087831 DHRSX 0.111781021 0.043564462
chrY 2354634 2354683 DHRSX 0.319536273 -0.010756246
chrY 2609031 2609080 CD99 0.150147449 0.065301529
chrY 59114746 59114795 IL9R
0.010313296 -0.02177692
chrY 59334093 59334142 SPRY3 0.014183914
0.010475217

Chromosome	Start	End	Feature	"PFC expression: 47,XXY vs Females"	"PFC expression: 47,XXY vs Males"
chrX	219773	219822	PLCXD1	0.498453822	0.152573833
chrX	221456	221505	GTPBP6	-0.605916557	-0.814257256
chrX	299374	299423	PPP2R3B	0.105274667	0.005419698
chrX	306290	306339	PPP2R3B	0.117336122	0.053870313
chrX	1428309	1428358	CSF2RA	0.093316341	0.070484869
chrX	1428310	1428359	CSF2RA	0.159181775	0.132357979
chrX	1428704	1428753	CSF2RA	-0.689173413	-0.403531324
chrX	1501338	1501387	IL3RA	0.014602501	0.009526626
chrX	1505329	1505378	SLC25A6	0.163333001	-0.072789037
chrX	1522143	1522193	ASMTL	0.451313426	0.233233339
chrX	1522214	1522263	ASMTL	0.46515281	0.096751368
chrX	1536914	1536963	ASMTL	0.468370608	0.260690284
chrX	2137782	2137831	DHRSX	0.080154998	0.083830162
chrX	2404634	2404683	DHRSX	0.286271325	0.184798919
chrX	2659031	2659080	CD99	0.355013728	0.123838213
chrX	3522660	3522709	PRKX	-0.22345308	-0.16616828
chrX	3522945	3522994	PRKX	-0.127451973	-0.108579088
chrX	6967201	6967250	HDHD1	0.091088595	0.372258095
chrX	7272357	7272406	STS	-0.066534782	-0.027757626
chrX	7867185	7867234	PNPLA4	0.324540158	0.428294006
chrX	9687610	9687659	TBL1X	-0.752491908	-0.607547509
chrX	10203332	10203381	CLCN4	0.076972117	-0.030987037
chrX	11778975	11779024	MSL3	-0.960196868	-0.730044402
chrX	11783794	11783843	MSL3	0.066164197	-0.054621166
chrX	11785862	11785911	MSL3	-0.121359275	-0.153405605
chrX	13730737	13730786	TRAPPC2	0.365794911	0.401843836
chrX	15802415	15802464	CA5B	0.010541703	0.048344886
chrX	15841166	15841215	ZRSR2	-0.321227532	-0.297458709
chrX	15844225	15844274	AP1S2	0.09753336	-0.110402956
chrX	15844477	15844526	AP1S2	0.696124874	0.475542322
chrX	16863144	16863193	RBBP7	0.188467421	0.06103031
chrX	20142985	20143034	EIF1AX	0.568669554	0.564767462
chrX	20148703	20148725	EIF1AX	0.327436972	0.246405573
chrX	20150300	20150326	EIF1AX	0.327436972	0.246405573
chrX	22010730	22010779	SMS	0.528235605	

					0.430648578
chrX	22010739	22010788	SMS	0.452806518	0.455733101
chrX	24095795	24095844	EIF2S3	-0.129310824	0.146463239
chrX	24231696	24231745	ZFX	0.203818222	0.42141856
chrX	38128919	38128968	RPGR	0.155075263	0.241599814
chrX	38156698	38156705	RPGR	0.077143363	0.07333064
chrX	38158209	38158250	RPGR	0.077143363	0.07333064
chrX	41092817	41092866	USP9X	0.452287758	0.591497403
chrX	41095608	41095657	USP9X	0.13756177	0.260377642
chrX	41208966	41209015	DDX3X	-0.153699628	0.286361005
chrX	44383336	44383385	FUNDC1	0.397891656	0.33742868
chrX	44970808	44970857	KDM6A	0.033479125	0.10033803
chrX	47074273	47074322	UBA1	0.295069239	0.407809539
chrX	49044434	49044483	SYP	0.357493692	-0.009317427
chrX	53401356	53401405	SMC1A	-0.097978188	-0.126118881
chrX	71493707	71493756	RPS4X	0.182617421	0.533386325
chrX	71495532	71495574	RPS4X	0.750567824	0.794225842
chrX	71496007	71496013	RPS4X	0.750567824	0.794225842
chrX	108884977	108885026	ACSL4	0.944629826	0.769984407
chrX	108911443	108911492	ACSL4	-0.152921423	-0.067022451
chrX	139865782	139865831	CDR1	-0.009190324	-0.045148058
chrX	155011740	155011789	SPRY3	0.011994772	0.036205739
chrX	155172999	155173048	VAMP7	0.372441263	0.222664262
chrY	169773	169822	PLCXD1	0.498453822	0.152573833
chrY	171456	171505	GTPBP6	-0.605916557	-0.814257256
chrY	249374	249423	PPP2R3B	0.105274667	0.005419698
chrY	256290	256339	PPP2R3B	0.117336122	0.053870313
chrY	1378309	1378358	CSF2RA	0.093316341	0.070484869

chrY	1378310	1378359	CSF2RA	0.159181775	0.132357979
chrY	1378704	1378753	CSF2RA	-0.689173413	-0.403531324
chrY	1451338	1451387	IL3RA	0.014602501	0.009526626
chrY	1455329	1455378	SLC25A6	0.163333001	-0.072789037
chrY	1472143	1472193	ASMTL	0.451313426	0.233233339
chrY	1472214	1472263	ASMTL	0.46515281	0.096751368
chrY	1486914	1486963	ASMTL	0.468370608	0.260690284
chrY	2087782	2087831	DHRSX	0.080154998	0.083830162
chrY	2354634	2354683	DHRSX	0.286271325	0.184798919
chrY	2609031	2609080	CD99	0.355013728	0.123838213
